# Psychosocial Correlates of Sedentary Behavior Reduction Readiness Relevant to Zero-Time Exercise Among Chinese Adults in Sedentary Occupations: Cross-Sectional Study

**DOI:** 10.2196/95707

**Published:** 2026-09-04

**Authors:** Ziyan Xiong, Xiangyuan Li, Lu Chai, Xiongqiang Duan, Xiang Hu, Yuan Xie, Li Cong

**Affiliations:** 1 Health Science Center Hunan Normal University Changsha, Hunan China; 2 Kiang Wu Nursing College of Macau Macau China

**Keywords:** zero-time exercise, sedentary behavior, occupational health, exercise motivation, self-efficacy, physical activity triggers, cross-sectional study

## Abstract

**Background:**

Prolonged sitting is an increasingly important public health concern, particularly in occupations that offer few opportunities for conventional exercise. Zero-time exercise (ZTEx) integrates brief muscle-engaging movements into daily routines without requiring dedicated time or equipment and may offer a practical way to interrupt prolonged sitting. However, the motivational, cognitive, and contextual factors associated with intention relevant to ZTEx adoption remain poorly understood.

**Objective:**

This study examined the association between exercise motivation and general sedentary behavior reduction intention, used as a zero-time exercise–related intention proxy (ZTEx-IP), and assessed model-implied indirect associations involving general self-efficacy and perceived physical activity triggers among Chinese adults in sedentary occupations.

**Methods:**

This cross-sectional survey included 790 adults working full-time in sedentary occupations across 20 provincial-level administrative divisions in China between May and September 2025. Eligible participants reported at least 6 hours of work-related sitting on a typical workday. Standardized questionnaires assessed exercise motivation, general self-efficacy, perceived physical activity triggers, and the ZTEx-IP. Multivariable regression and restricted cubic splines examined graded and nonlinear associations between exercise motivation and the ZTEx-IP. PROCESS Models 4 and 6 estimated covariate-adjusted direct and model-implied indirect associations using 5000 bootstrap resamples.

**Results:**

Higher exercise motivation scores were associated with higher ZTEx-IP scores. Compared with participants in the lowest motivation tertile, those in the highest tertile had an adjusted score difference of 8.49 points (unstandardized regression coefficient, B=8.49, 95% CI 6.43-10.60; *P*<.001), and the association was nonlinear (*P* for nonlinearity<.001). In the prespecified ordered model, exercise motivation retained a direct association with the ZTEx-IP (B=0.167, 95% CI 0.088-0.246). Model-implied indirect associations were observed through general self-efficacy (B=0.104, 95% CI 0.057-0.155), perceived physical activity triggers (B=0.157, 95% CI 0.110-0.205), and their prespecified ordering (B=0.025, 95% CI 0.013-0.039). Perceived triggers accounted for a larger increment in explained variance than general self-efficacy (Δ*R*²=0.054 vs 0.033), and the adjusted outcome model explained 37.6% of the variance in ZTEx-IP scores (*R*²=0.376).

**Conclusions:**

Among Chinese adults in sedentary occupations, exercise motivation was positively associated with the ZTEx-IP, with model-implied indirect associations involving general self-efficacy and perceived physical activity triggers. The statistical ordering of these associations does not establish that the constructs develop sequentially over time. The comparatively larger trigger-related estimate identifies contextual cue salience as a candidate mechanism for prospective testing but does not demonstrate intervention effectiveness. These hypothesis-generating findings provide a basis for workplace trials of contextually adapted, multicomponent strategies that evaluate actual ZTEx frequency, objectively measured sitting interruptions, adherence over time, and workplace acceptability.

## Introduction

Sedentary behavior is defined as any waking behavior characterized by an energy expenditure of 1.5 metabolic equivalents (METs) or less while sitting, reclining, or lying down [1]. Prolonged sitting has been associated with increased risks of dementia, cardiovascular disease, metabolic disorders, and all-cause mortality [2-4]. In China, approximately one-quarter of adults report sitting for more than 6 hours per day, and prolonged occupational sitting is increasingly common [5]. Given that work organization can limit opportunities for conventional exercise, feasible strategies for interrupting sitting without substantially disrupting routine tasks are needed. Zero-time exercise (ZTEx) refers to brief, simple, muscle-engaging movements performed as part of an existing daily activity, without a separate exercise session, specialized equipment, or additional scheduled time [6]. Examples include seated leg raises during desk work and shoulder retraction while typing. Unlike accumulated exercise, which divides physical activity into several discrete bouts, ZTEx is performed alongside occupational or household tasks. This feature may be particularly relevant to workers whose schedules or job demands limit participation in conventional exercise. Evidence that brief, lifestyle-integrated activity can improve cardiometabolic and functional outcomes further supports the potential value of incorporating movement into sedentary routines [7-9].

Research on ZTEx has largely examined its feasibility and preliminary health outcomes [10], while less is known about the psychosocial factors associated with intention to adopt it. Motivation, self-regulation, and perceived capability are established correlates of physical activity [11,12], but ZTEx may also depend on whether brief movement is perceived as feasible within an ongoing task. Workplace conditions such as time pressure, environmental cues, task structure, and organizational norms may therefore be especially relevant to this form of lifestyle-integrated activity [13]. Yet these factors have generally been studied separately, leaving their joint associations with intention relevant to ZTEx adoption unclear.

General sedentary behavior reduction intention and readiness to adopt ZTEx are conceptually related but not equivalent. Their overlap lies in the evaluation of interrupting sitting, perceived social expectations regarding such interruptions, perceived control over doing so, and willingness to change sedentary routines [14]. However, reducing sedentary behavior is a broad behavioral goal that may be pursued through standing, walking, conventional activity breaks, or ZTEx. ZTEx is a more specific means of pursuing this goal through brief muscle-engaging movements embedded within ongoing activities. The relationship between the constructs can therefore be understood as increasing behavioral specificity, from a general intention to reduce sedentary behavior, through willingness to interrupt a sitting episode and readiness to integrate movement into an ongoing activity, to performance of a defined ZTEx behavior in a particular context [15]. The zero-time exercise–related intention proxy (ZTEx-IP) is positioned at the broader end of this framework because it reflects general willingness and perceived control relevant to changing sedentary routines without specifying ZTEx as the means of change. ZTEx-specific readiness additionally involves beliefs about performing particular movements, knowledge of appropriate techniques, movement-specific confidence and outcome expectations, perceived feasibility and compatibility with ongoing tasks, and workplace appropriateness [16,17].

Because ZTEx is embedded within an ongoing activity, it must be initiated while another goal, such as completing a work task, is already active. This feature guided the selection of exercise motivation, general self-efficacy, and perceived physical activity triggers. Exercise motivation reflects the quality and internalization of the value placed on movement. This motivational quality is relevant when movement competes with the immediate convenience of sitting, habitual behavior, and task demands. Self-determination theory provides a theoretical account of this variation in motivational quality by distinguishing autonomous and controlled forms of regulation [18]. General self-efficacy represents a broader agentic resource for managing obstacles and competing demands. Social cognitive theory supports its inclusion because efficacy beliefs influence goal pursuit, effort, and persistence in the presence of impediments [19]. Perceived physical activity triggers represent the salience of cues and facilitators that make otherwise overlooked movement opportunities noticeable during work. This is particularly relevant when employees remain seated because their attention is absorbed by ongoing tasks. Workplace evidence identifies competing motivations, limited perceived control, task and organizational constraints, and missed or ignored cues as barriers to interrupting sitting. The three variables were therefore selected to represent motivational regulation, generalized perceived agency, and cue salience within the specific demands of lifestyle-integrated movement. They were not intended to capture all determinants of ZTEx adoption.

The capability, opportunity, motivation-behavior (COM-B) model and the Fogg behavior model provide complementary frameworks for organizing these factors at different explanatory levels rather than functioning as competing models. COM-B provides a system-level account in which behavior depends on motivation, capability, and opportunity [20,21]. At a broad conceptual level, exercise motivation relates to motivational resources, general self-efficacy to perceived psychological capability, and perceived physical activity triggers to the salience of cues and facilitators that signal opportunities for action. Nevertheless, the constructs measured in this study are not direct operational equivalents of these framework components. For ZTEx, capability also encompasses technique-specific knowledge and movement skills, whereas opportunity encompasses task compatibility and workplace norms. The Fogg behavior model provides a more immediate account of behavioral initiation: a prompt is expected to elicit action only when sufficient motivation and ability are present at the same moment [22]. Thus, self-determination theory provides a theoretical account of motivational quality, social cognitive theory supports the role of generalized perceived agency, COM-B situates these factors within broader behavioral conditions, and the Fogg behavior model highlights the conditional role of prompts at the moment of action.

Therefore, this study aimed to examine the association between exercise motivation and the ZTEx-IP among Chinese adults in sedentary occupations and to evaluate model-implied indirect associations involving general self-efficacy and perceived physical activity triggers. We also characterized participants’ sedentary behavior and physical activity patterns and examined whether the association between exercise motivation and the ZTEx-IP was graded or nonlinear. We hypothesized that higher exercise motivation would be positively associated with the ZTEx-IP and that the fitted models would yield positive indirect associations involving general self-efficacy and perceived physical activity triggers.

## Methods

### Design

This cross-sectional online survey examined contemporaneous associations among exercise regulation, general self-efficacy, perceived physical activity triggers, and the ZTEx-IP. Theory-informed parallel and ordered indirect-association analyses were used to quantify model-implied indirect associations. Because all variables were measured at the same time, these analyses were not intended to establish temporal or causal mediation. Reporting followed the STROBE (Strengthening the Reporting of Observational Studies in Epidemiology) checklist (Multimedia Appendix 1).

### Setting and Sample

Data were collected through an online questionnaire in China from May to September 2025. The study included participants from 20 of China’s 34 provincial-level administrative divisions, covering eastern, central, and western regions. Recruitment used a multichannel convenience-sampling strategy. Recruitment information was distributed through workplace WeChat (Tencent Holdings Limited) groups, occupation-related online communities, and workplace or institutional contacts. Individual recruitment source was not recorded; therefore, channel-specific recruitment proportions could not be calculated. Self-reported work-unit information indicated participation from public-service institutions, enterprises, and other workplace settings. Organization size and ownership were not systematically recorded. A standardized recruitment notice described the study purpose, eligibility criteria, voluntary participation, confidentiality, approximate completion time, and the right to withdraw before submission. Contacts only forwarded the notice and questionnaire link and had no access to individual responses. The electronic questionnaire was administered through Wenjuanxing and accessed via a web link or QR code. Participants provided electronic informed consent before entering the survey. A standardized definition of ZTEx was presented before the ZTEx-related items. The inclusion criteria required (1) adults aged ≥18 years; (2) employed full-time in sedentary occupations, operationally defined as jobs predominantly involving seated work for ≥6 hours per working day with low energy expenditure (≤1.5 METs), consistent with the Sedentary Behaviour Research Network definition and commonly used cutoffs in occupational sedentary epidemiology; and (3) able to provide informed consent. Exclusion criteria eliminated individuals on long-term leave, with cognitive impairment affecting comprehension, or participating in concurrent behavioral intervention programs.

Of the 820 submitted questionnaires, 30 were excluded because missing data precluded calculation of at least one core scale score or analysis variable, leaving 790 questionnaires for analysis. Thus, 96.3% (790/820) of submitted questionnaires were included; this percentage represents analytic inclusion rather than a response rate because the number of individuals who received or viewed the recruitment information was unavailable. No item-level imputation was performed. Before data collection, sample-size planning used a pragmatic ratio of 10 to 20 participants per anticipated model parameter. Allowing 20% for unusable questionnaires yielded a recruitment target of 325 to 650 participants. Because sample-size requirements for indirect associations depend on path magnitudes and model characteristics [23,24], this heuristic was not regarded as a model-specific power calculation. A subsequent scenario-based Monte Carlo analysis of sample adequacy is reported in Multimedia Appendix 2.

### Data Collection Tools

#### General Information Questionnaire

The questionnaire was developed based on a literature review and study goals, covering 17 items and related sociodemographic and work characteristics. Variables included sex, age, educational attainment, monthly income, occupation category, job tenure, cumulative sedentary hours per day, musculoskeletal discomfort, recent health examination, chronic disease history, physical activity duration, exercise frequency and duration, use of wearable devices, and familiarity with ZTEx. For descriptive reporting, physical activity was separated based on context and intent. Incidental physical activity was defined as physical activity accumulated during work-related activities, household duties, transportation, and other routine daily activities, other than physical activity undertaken during a planned exercise session. Structured exercise was defined as a planned, intentional, and repetitive form of physical activity that was undertaken specifically with the aim of improving or maintaining health or physical fitness [25]. Frequency and duration were recorded separately for these 2 activity categories. Before answering the familiarity item, participants were shown the standardized definition of ZTEx. Familiarity was assessed with the single self-report item, “How familiar are you with zero-time exercise?” Responses were categorized as “not familiar,” “somewhat familiar,” or “very familiar” and retained in these categories for descriptive analyses. For the exploratory familiarity-restricted analysis, “somewhat familiar” and “very familiar” were combined as “at least somewhat familiar,” whereas participants who selected “not familiar” were excluded.

#### Adult Sedentary Behavior Reduction Intention Questionnaire

The Adult Sedentary Behavior Reduction Intention Questionnaire was developed by Ye et al [26] from the theory of planned behavior to measure adults’ intention to reduce sedentary behavior. It contains 4 dimensions: behavioral intention, attitude, subjective norms, and perceived behavioral control. The scale has 16 items, each rated on a 5-point Likert scale (1 to 5), with higher values showing greater intention to reduce sedentary behavior. In the absence of a validated ZTEx-specific intention measure, the questionnaire total score was operationalized as the ZTEx-IP. This operationalization was based on the conceptual overlap between reducing sedentary behavior and incorporating brief, routine-integrated movements into otherwise sedentary daily activities. However, the questionnaire assesses general sedentary behavior reduction intention rather than intention to perform ZTEx specifically; therefore, the outcome should be interpreted as a proxy indicator rather than a direct measure of ZTEx-specific intention. The validation study reported excellent internal consistency (Cronbach α=0.967) and strong construct validity, with subscale α ranging from 0.911 to 0.950. In this study, Cronbach α for this scale was 0.953.

#### Behavioral Regulation in Exercise Questionnaire

The Behavioral Regulation in Exercise Questionnaire (BREQ-2) was developed by Markland and Tobin in 2004 to assess exercise motivation within self-determination theory [27]. It includes 5 dimensions: amotivation, external regulation, introjected regulation, identified regulation, and intrinsic motivation. The Chinese version contains 18 items rated on a 5-point Likert scale from 0 to 4. In this study, amotivation and external regulation items were reverse scored before all items were summed to create a composite score; higher scores represented a more self-determined motivational profile. The BREQ-2 captures relatively stable forms of behavioral regulation and motivational internalization rather than the momentary intensity of motivation immediately before an action, as conceptualized in the Fogg behavior model. Liu et al [28] translated and validated the Chinese version in 2015, reporting Cronbach α values ranging from 0.72 to 0.85 and good construct validity. Cronbach α was 0.882 in this study.

#### General Self-Efficacy Scale

General Self-Efficacy Scale (GSES) was developed by Schwarzer and Jerusalem in 1995 to assess individuals’ confidence in coping with challenges and controlling demanding situations [29]. The unidimensional scale contains 10 items rated from 1 to 4, with higher scores indicating greater general self-efficacy. Wang et al [30] translated and validated the Chinese version in 2001 [30], reporting Cronbach α=0.87, test-retest reliability *r*=0.83, and split-half reliability *r*=0.82. Cronbach α was 0.938 in this study. The GSES assesses generalized rather than ZTEx-specific self-efficacy and was therefore not treated as a direct operationalization of behavior-specific capability in the COM-B model. Instead, it was used as a theoretically relevant measure of generalized perceived agency within the proposed psychosocial model.

#### Physical Activity Triggers Questionnaire

The Physical Activity Triggers Questionnaire (PATQ) was developed by Wang and Kang in 2022 to assess physical activity-related triggers [31]. It consists of 3 dimensions: spark, signal, and facilitator. The PATQ has 14 items measured on a 5-point Likert scale (1=strongly disagree, 5=strongly agree). The PATQ captures perceived exposure or receptivity to physical activity cues in daily life rather than brief prompts occurring immediately before a specific action. It was therefore used to examine cue-related associations with the other study variables. The original validation among Chinese university students reported strong internal consistency (Cronbach α=0.925), split-half reliability of 0.821, test-retest reliability of 0.860, and acceptable construct validity. Cronbach α was 0.934 in this study.

### Data Analysis

All statistical analyses were conducted using R (version 4.5.1; R Core Team) and IBM SPSS Statistics version 29.0 with the PROCESS macro. Scale-score distributions were assessed using the Shapiro-Wilk test. Categorical variables were summarized as frequencies and percentages, and scale scores as medians and IQRs (25th-75th percentiles). Internal consistency was evaluated using Cronbach α. Differences in the ZTEx-IP across participant characteristics were examined using Mann-Whitney U tests or Kruskal-Wallis H tests, as appropriate. The Benjamini-Hochberg procedure was applied to the 15 comparisons between-group comparisons; unadjusted and adjusted *P* values are reported in Multimedia Appendix 3. Spearman rank correlations were used to examine associations among the 4 focal scores. Harman’s unrotated single-factor analysis of the 58 core questionnaire items was used as a limited diagnostic for common method variance.

The adjustment set was defined using a combination of substantive considerations and nominal empirical screening. Candidate variables represented 4 relevant domains. Age and monthly income represented sociodemographic context; incidental physical activity duration and structured exercise frequency and duration represented existing movement patterns; wearable-device use represented potential exposure to activity monitoring and digital cues; and ZTEx familiarity represented prior awareness of the target strategy. The socioeconomic and activity-related rationale was informed by previous literature [32], whereas wearable-device use and ZTEx familiarity were included on substantive grounds because cue exposure and prior knowledge could be related to the focal study variables. To obtain a parsimonious adjustment set for this exploratory analysis, candidate variables meeting the nominal significance criterion (α=.05) in the initial unadjusted comparisons were retained. The resulting set comprised age, monthly income, incidental physical activity duration, structured exercise frequency, structured exercise duration, wearable-device use, and ZTEx familiarity. Monthly income met the nominal screening criterion before multiplicity adjustment (unadjusted *P*=.04), although the Benjamini-Hochberg–adjusted comparison was not statistically significant (adjusted *P*=.07). The Benjamini-Hochberg procedure was used only for descriptive multiplicity control and did not redefine the adjustment set. The selected variables were treated as adjustment covariates rather than empirically established confounders. Age, income, incidental physical activity duration, structured exercise frequency and duration, and ZTEx familiarity were entered as ordinal category scores, whereas wearable-device use was entered as a binary indicator. Ordinary least squares regression was used to examine the association between exercise motivation and the ZTEx-IP. Motivation was modeled using sample-specific tertiles, with the lowest tertile as the reference and an ordinal term to test for trend. The continuous association was examined using a restricted cubic spline with knots at motivation scores of 33, 48, 54, and 72 and a reference score of 51; overall and nonlinear associations were tested.

Model-implied indirect associations were estimated using PROCESS Models 4 and 6. Model 4 specified general self-efficacy and perceived physical activity triggers as parallel intermediate variables, whereas Model 6 imposed the prespecified theory-informed ordering of exercise motivation, general self-efficacy, perceived physical activity triggers, and the ZTEx-IP to estimate the corresponding model-implied indirect association. The same covariates were included in every component equation. Direct, specific indirect, total indirect, and total associations were estimated using 5000 nonparametric percentile bootstrap resamples. A 95% bootstrap CI excluding 0 was considered statistical evidence of an indirect association. Unstandardized coefficients were the primary estimates, with standardized coefficients additionally reported for the focal associations. Model *R*² and overall *F* statistics were reported. The incremental contribution of each intermediate variable was assessed using Δ*R*² and Cohen *f*². Because all variables were measured concurrently, the indirect associations were interpreted as statistical decompositions of cross-sectional associations rather than evidence of temporal or causal mediation. Model assumptions were assessed using variance inflation factors and residual diagnostics, with HC3 heteroscedasticity-consistent SEs examined as a robustness analysis. Three theoretically motivated sensitivity analyses were prioritized: alternative outcome definitions using the behavioral intention subscale and a score excluding perceived behavioral control items, restriction to participants who were at least somewhat familiar with ZTEx, and an alternative ordering of general self-efficacy and perceived physical activity triggers. Additional regression diagnostics and technical robustness analyses are reported in Multimedia Appendices 4 and 5. Bootstrap-based sensitivity analyses used 5000 resamples. All sensitivity analyses were considered exploratory, and no additional multiplicity adjustment was applied.

### Ethical Considerations

The study was conducted in accordance with the Declaration of Helsinki, and ethical approval was granted by the Ethics Committee of Hunan Normal University (2025889). Before beginning the online questionnaire, participants received information about the study purpose, voluntary participation, data confidentiality, and their right to decline participation or withdraw before submission without penalty. All participants provided electronic informed consent before proceeding to the survey items. Participants received no financial or material compensation for participation. To protect participant privacy and confidentiality, the analytic dataset was deidentified before analysis by removing information that could identify individual participants or participating institutions. Results were reported in aggregate, data were used solely for the purposes of this study, and access was restricted to the research team.

## Results

### Descriptive Statistics

Of the 820 online questionnaire submissions received, 790 met the completeness requirements described in the “Methods” section and were included in the analyses, corresponding to an analytic inclusion proportion of 96.3% (790/820). Table 1 summarizes participants’ sociodemographic, occupational, and behavioral characteristics and their median ZTEx-IP scores. The sample was relatively balanced by sex, with female participants accounting for 56.6% (447/790), and 75.4% (596/790) of participants were younger than 40 years. Education and research professionals formed the largest occupational group (297/790, 37.6%). Most participants reported middle-income levels and had completed higher education.

**Table 1:** Demographic, occupational, and behavioral characteristics and ZTEx-IP^a^ scores among participants (n=790)^b^.

Variables		n (%)	Median (IQR)	BH^c^-adjusted *P*	
**Sex**					.80
	Male	343 (43.4)	58 (48-67)		
	Female	447 (56.6)	59 (51-64)		
**Age (y)**					.04
	<30	324 (41)	58 (49-64)		
	30-40	272 (34.4)	59 (50-66)		
	41-50	128 (16.2)	62 (52-69)		
	51-60	52 (6.6)	61 (50-65)		
	>60	14 (1.8)	52 (48-63)		
**Educational attainment**					.38
	High school diploma or below	272 (34.4)	58 (49-66)		
	Undergraduate degree	400 (50.6)	60 (50-65)		
	Master’s degree or above	118 (14.9)	60 (53-66)		
**Average monthly income (RMB)^d^**					.07
	<2000	98 (12.4)	55 (48-64)		
	2000-5000	384 (48.6)	59 (50-65)		
	5000-10,000	247 (31.3)	59 (51-66)		
	>10,000	61 (7.7)	61 (52-70)		
**Occupation category**					.14
	Office-based workers	161 (20.4)	57 (48-64)		
	Education and research professionals	297 (37.6)	60 (51-66)		
	IT and technical professionals	220 (27.8)	60 (53-67)		
	Drivers and transport personnel	112 (14.2)	58 (48-65)		
**Job tenure (y)**					.20
	<5	283 (35.8)	58 (49-64)		
	5-15	289 (36.6)	59 (51-66)		
	15-25	121 (15.3)	58 (48-68)		
	>25	97 (12.3)	63 (53-68)		
**Cumulative daily sedentary duration (h)**					.37
	<8	555 (70.3)	60 (51-66)		
	8-10	166 (21)	58 (49-64)		
	>10	69 (8.7)	56 (48-67)		
**Musculoskeletal discomfort**					.37
	No	137 (17.3)	57 (48-67)		
	Mild (occasional soreness)	535 (67.7)	60 (51-65)		
	Moderate (affecting work)	93 (11.8)	58 (48-66)		
	Severe (requiring treatment)	25 (3.2)	55 (47-65)		
**Health examination within the past 6 months**					.32
	Yes	466 (59)	59 (50-67)		
	No	324 (41)	59 (51-64)		
**Chronic disease**					.25
	Yes	217 (27.5)	60 (52-67)		
	No	573 (72.5)	59 (50-65)		
**Cumulative duration of daily** **incidental physical activity** **(min)**					.001
	<30	190 (24.1)	55 (48-64)		
	30-60	328 (41.5)	59 (52-65)		
	60-90	114 (14.4)	62 (51-69)		
	>90	158 (20)	60 (52-69)		
**Frequency of** **structured exercise** **(sessions/wk)**					<.001
	0	255 (32.3)	53 (48-63)		
	1-2	366 (46.3)	60 (53-66)		
	3-4	117 (14.8)	62 (53-69)		
	≥5	52 (6.6)	68 (60-78)		
**Average duration per structured exercise session (min)**					<.001
	<10	246 (31.1)	53 (48-63)		
	10-30	282 (35.7)	59 (52-65)		
	30-60	180 (22.8)	62 (56-69)		
	>60	82 (10.4)	64 (49-73)		
**Use of wearable health-tracking devices**					.001
	Yes	263 (33.3)	61 (53-67)		
	No	527 (66.7)	58 (48-65)		
**Familiarity with ZTEx^e^**					.04
	Not familiar	603 (76.3)	58 (49-65)		
	Somewhat familiar	154 (19.5)	61 (53-67)		
	Very familiar	33 (4.2)	59 (53-80)		

^a^ZTEx-IP: zero-time exercise–related intention proxy.

^b^Incidental physical activity refers to movement accumulated through occupational duties, household tasks, transportation, and other routine daily activities outside planned exercise sessions. Structured exercise refers to planned, intentional, and repetitive physical activity undertaken to improve or maintain health or physical fitness. The presented *P* values were adjusted using the Benjamini-Hochberg procedure. Detailed test statistics, unadjusted *P* values, and Benjamini-Hochberg–adjusted results are provided in Multimedia Appendix 3.

^c^Benjamini-Hochberg.

^d^The 2025 average exchange rate was US $1=RMB 7.1875.

^e^ZTEx: zero-time exercise.

The median ZTEx-IP score was 59 (IQR 50-65.75). After Benjamini-Hochberg adjustment, differences remained statistically significant across age groups (adjusted *P*=.04), incidental physical activity duration (adjusted *P*=.001), structured exercise frequency (adjusted *P*<.001), structured exercise duration (adjusted *P*<.001), wearable-device use (adjusted *P*=.001), and ZTEx familiarity (adjusted *P*=.04). No statistically significant differences were observed by sex, educational attainment, occupation, job tenure, daily sedentary duration, musculoskeletal discomfort, health examination in the previous 6 months, or chronic disease status (all adjusted *P*>.05).

### Correlation Analysis

Spearman rank correlations were used to examine associations among exercise motivation, general self-efficacy, perceived physical activity triggers, and the ZTEx-IP (Table 2). All correlations were positive and statistically significant (all *P*<.001). Exercise motivation was positively correlated with general self-efficacy (ρ=0.347), perceived physical activity triggers (ρ=0.539), and the ZTEx-IP (ρ=0.371). General self-efficacy was positively correlated with perceived physical activity triggers (ρ=0.349) and the ZTEx-IP (ρ=0.378). Perceived physical activity triggers showed the strongest correlation with the ZTEx-IP (ρ=0.420).

**Table 2:** Spearman rank correlations among exercise motivation, general self-efficacy, perceived physical activity triggers, and the ZTEx-IP^a^ (n=790)^b^.

Variable	Median (IQR)	1	2	3	4
Exercise motivation	51 (44-56)	—^c^			
General self-efficacy	29 (26-30)	.347^d^	—		
Perceived physical activity triggers	36 (28-43)	.539^d^	.349^d^	—	
ZTEx-IP	59 (50-65.75)	.371^d^	.378^d^	.420^d^	—

^a^ZTEx-IP: zero-time exercise–related intention proxy.

^b^Scale scores are presented as median (IQR). Values in the lower triangle are Spearman rank correlation coefficients (ρ). The ZTEx-IP was operationalized using the total score of the Adult Sedentary Behavior Reduction Intention Questionnaire.

^c^Self-correlations, which were not estimated.

^d^*P*<.001.

### Association Between Exercise Motivation and ZTEx-IP

**Figure 1:**
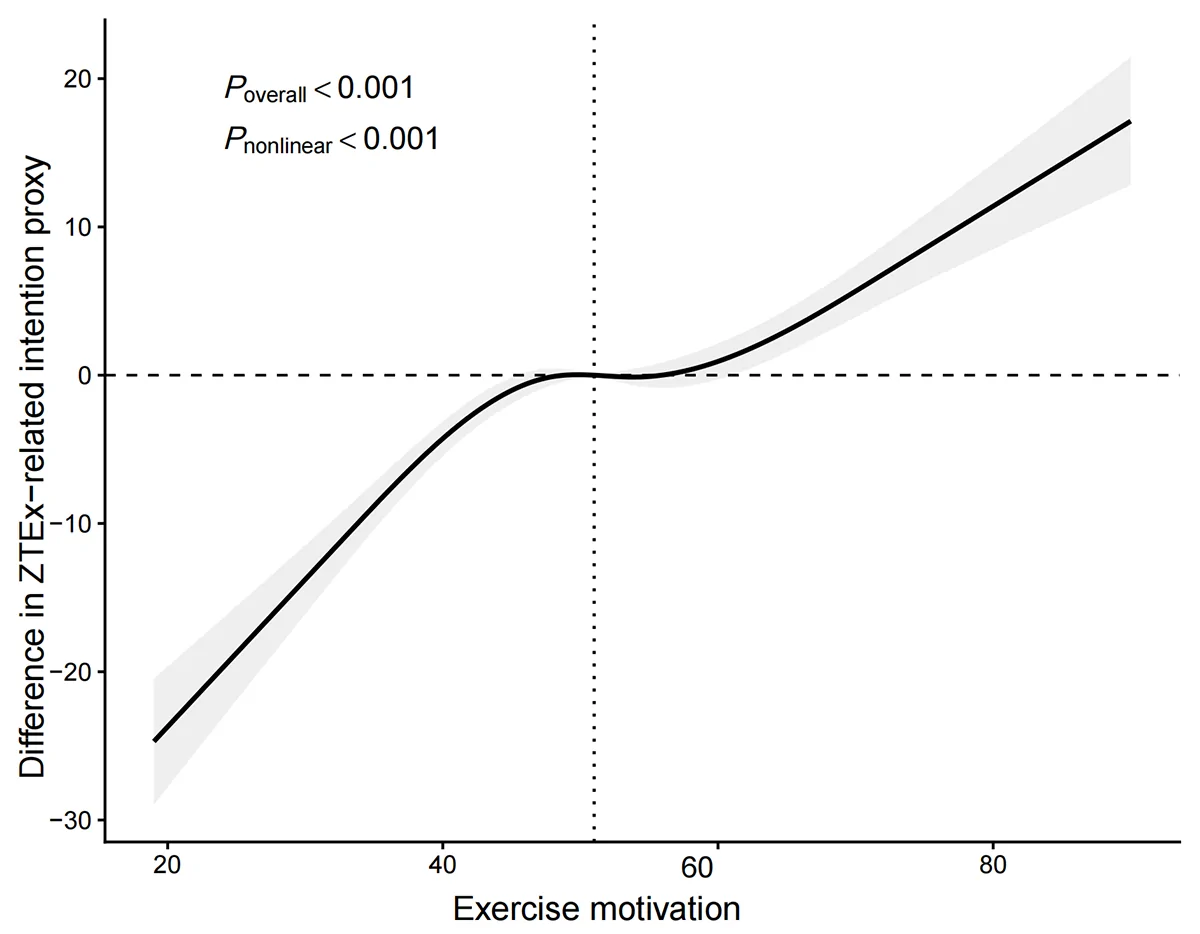
Restricted cubic spline analysis of the association between exercise motivation and the zero-time exercise–related intention proxy (ZTEx-IP) among Chinese adults in sedentary occupations. The solid line shows the multivariable-adjusted difference in the ZTEx-IP, and the shaded band shows the 95% CI. The vertical dotted line marks the reference motivation score of 51, at which the adjusted difference was set to 0; the horizontal dashed line indicates no difference from that reference. Negative values indicate lower adjusted ZTEx-IP scores relative to the reference and do not imply an inverse overall association. The model was adjusted for age, income, incidental physical activity duration, structured exercise frequency, structured exercise duration, wearable-device use, and zero-time exercise familiarity. Both the overall and nonlinear associations were statistically significant (*P*<.001 for each). ZTEx: zero-time exercise.

Participants were categorized into tertiles according to their exercise motivation scores. In the unadjusted model, compared with participants in the lowest motivation tertile, those in the middle and highest tertiles had higher ZTEx-IP scores, with B coefficients of 5.58 (95% CI 3.60-7.56; *P*<.001) and 10.60 (95% CI 8.63-12.60; *P*<.001), respectively. After adjustment for age, income, incidental physical activity duration, structured exercise frequency, structured exercise duration, wearable-device use, and ZTEx familiarity, the corresponding B coefficients were 4.64 (95% CI 2.69-6.60; *P<*.001) and 8.49 (95% CI 6.43-10.60; *P*<.001), respectively (Multimedia Appendix 6). A significant positive trend was observed across increasing motivation tertiles (adjusted B for trend=4.25, 95% CI 3.22-5.28; *P*<.001). When exercise motivation was analyzed as a continuous variable, restricted cubic spline analysis showed a significant overall association with the ZTEx-IP (*P* overall<.001), with evidence of nonlinearity (*P* nonlinear<.001; Figure 1). Using an exercise motivation score of 51 as the reference value, the spline curve showed an overall increasing pattern, although the magnitude of the association varied across the motivation score range.

### Parallel Indirect-Association Analysis (PROCESS Model 4)

Before the indirect-association analyses, common method variance was assessed using Harman’s single-factor test. All 58 core items from the BREQ-2, GSES, PATQ, and Adult Sedentary Behavior Reduction Intention Questionnaire were included. The first unrotated factor accounted for 31.93% of the total variance. This result did not indicate a predominant single factor, although the test cannot rule out common method bias. Full results are reported in Multimedia Appendix 7.

**Figure 2:**
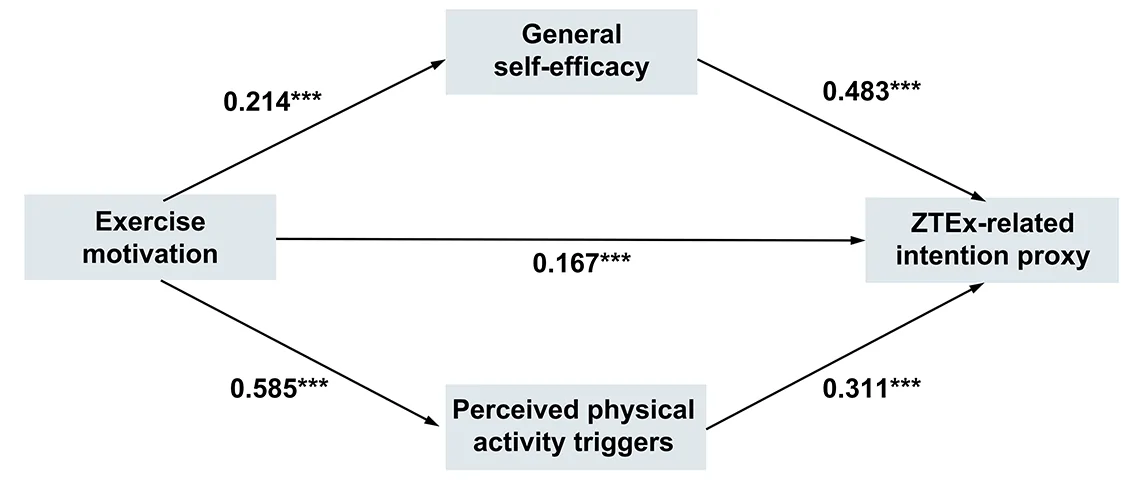
Adjusted parallel indirect-association model involving exercise motivation, general self-efficacy, perceived physical activity triggers, and the zero-time exercise–related intention proxy. Values are unstandardized regression coefficients. The model was adjusted for age, income, wearable-device use, incidental physical activity duration, structured exercise frequency, structured exercise duration, and zero-time exercise familiarity. ZTEx: zero-time exercise. ***P<.001.

A parallel indirect-association model was fitted to estimate model-implied indirect associations involving general self-efficacy and perceived physical activity triggers. The model was adjusted for age, income, incidental physical activity duration, structured exercise frequency, structured exercise duration, wearable-device use, and ZTEx familiarity. The adjusted parallel indirect-association model is illustrated in Figure 2. Exercise motivation was positively associated with both general self-efficacy (B=0.214, SE 0.015, 95% CI 0.184-0.245; *P*<.001) and perceived physical activity triggers (B=0.585, SE 0.031, 95% CI 0.524-0.645; *P*<.001). In the outcome model, both general self-efficacy (B=0.483, SE 0.075, 95% CI 0.336-0.631; *P*<.001) and perceived physical activity triggers (B=0.311, SE 0.038, 95% CI 0.237-0.386; *P*<.001) were positively associated with the ZTEx-IP after adjustment for exercise motivation and covariates. The direct association between exercise motivation and the ZTEx-IP remained significant (B=0.167, SE 0.04, 95% CI 0.088-0.246; *P*<.001). Bootstrap analysis with 5000 resamples yielded nonzero indirect-association estimates involving general self-efficacy (B=0.104, bootstrap SE (BootSE)=0.026, 95% CI 0.056-0.158) and perceived physical activity triggers (B=0.182, BootSE=0.026, 95% CI 0.129-0.233). The total indirect association was also significant (B=0.286, BootSE=0.034, 95% CI 0.220-0.353). The adjusted outcome model explained 37.6% of the variance in the ZTEx-IP. Detailed adjusted Model 4 results are provided in Multimedia Appendix 8.

### Ordered Indirect-Association Analysis (PROCESS Model 6)

PROCESS Model 6 was fitted to estimate model-implied indirect associations under the prespecified theory-informed ordering of exercise motivation, general self-efficacy, perceived physical activity triggers, and the ZTEx-IP. As shown in Table 3, exercise motivation was positively associated with both general self-efficacy (standardized regression coefficient, β=0.460, *P*<.001) and perceived physical activity triggers (β=0.487, *P*<.001), and general self-efficacy was positively associated with perceived physical activity triggers (β=0.170, *P*<.001). These coefficients were compatible with the prespecified statistical ordering, but they do not establish temporal precedence among the constructs. In the full model predicting the ZTEx-IP, exercise motivation (β=0.161, *P*<.001), general self-efficacy (β=0.216, *P*<.001), and perceived physical activity triggers (β=0.310, *P*<.001) all remained statistically significant. The adjusted outcome model explained 37.6% of the variance in the ZTEx-IP (*R*²=0.376, *F*_10,779_=46.995; *P*<.001). Compared with reduced models excluding each intermediate variable separately, general self-efficacy contributed an additional 3.3% of explained variance (Δ*R*²=0.033, Cohen *f*²=0.053), whereas perceived physical activity triggers contributed an additional 5.4% (Δ*R*²=0.054, Cohen *f*²=0.087). Complete model diagnostics and secondary technical robustness analyses are reported in Multimedia Appendix 4.

**Table 3:** Component equations for the prespecified ordered model (n=790)^a^.

Regression		Model index		Coefficients	
Dependent variable	Independent variable	*R*²	*F* test (*df*)	β	*t* test (*df*)
General self-efficacy	Exercise motivation	0.269	35.997 (8, 781)^b^	0.460	13.876 (781)^b^
Perceived physical activity triggers	Exercise motivation	0.436	66.928 (9, 780)^b^	0.487	14.960 (780)^b^
Perceived physical activity triggers	General self-efficacy	—^c^	—	0.170	5.404 (780)^b^
ZTEx-IP^d^	Exercise motivation	0.376	46.995 (10, 779)^b^	0.161	4.150 (779)^b^
ZTEx-IP	General self-efficacy	—	—	0.216	6.428 (779)^b^
ZTEx-IP	Perceived physical activity triggers	—	—	0.310	8.239 (779)^b^

^a^All 3 equations were adjusted for age, income, incidental physical activity duration, structured exercise frequency, structured exercise duration, wearable-device use, and ZTEx familiarity. β denotes a standardized coefficient. The focal continuous variables were analyzed using their original scale scores and standardized for presentation in this table. Ordered categorical covariates were entered as ordinal category scores, and wearable-device use was entered as a binary indicator.

^b^*P*<.001.

^c^Not applicable.

^d^ZTEx-IP: zero-time exercise–related intention proxy.

### Bootstrap Estimation of the Ordered Indirect-Association Model

**Figure 3:**
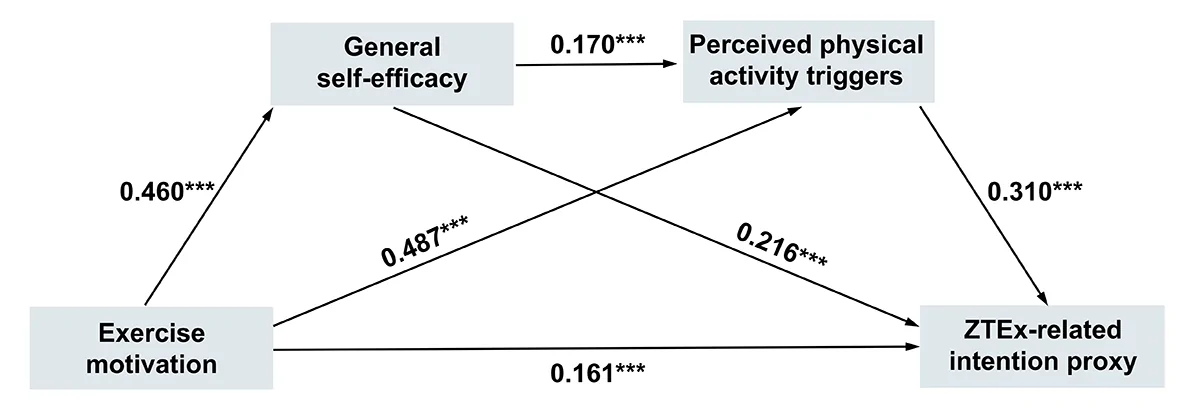
Standardized coefficient diagram for the prespecified ordered model involving exercise motivation, general self-efficacy, perceived physical activity triggers, and the zero-time exercise–related intention proxy. Coefficients were adjusted for age, income, incidental physical activity duration, structured exercise frequency, structured exercise duration, wearable-device use, and zero-time exercise familiarity. Arrows indicate the imposed model specification and do not establish temporal sequence. ZTEx: zero-time exercise. ***P<.001.

A percentile bootstrap analysis with 5000 samples was conducted to evaluate model-implied indirect associations under the prespecified ordering. As shown in Table 4, the total indirect association between exercise motivation and the ZTEx-IP was B=0.286 (BootSE=0.034, 95% CI 0.218-0.352). The three specific estimates involved (1) general self-efficacy alone (B=0.104, BootSE=0.025, 95% CI 0.057-0.155), (2) perceived physical activity triggers alone (B=0.157, BootSE=0.024, 95% CI 0.110-0.205), and (3) the prespecified ordered combination of general self-efficacy and perceived physical activity triggers (B=0.025, BootSE=0.007, 95% CI 0.013-0.039). All bootstrap CIs excluded 0. The direct association between exercise motivation and the ZTEx-IP also remained statistically significant (B=0.167, SE=0.040, 95% CI 0.088-0.246). Both the direct association and the model-implied indirect association estimates therefore differed from 0. The observed statistical pattern was consistent with the theory-informed ordering used in PROCESS Model 6. However, the cross-sectional estimates cannot determine whether self-efficacy precedes trigger perception, whether trigger perception enhances self-efficacy, or whether both are influenced by another unmeasured factor. Figure 3 presents standardized coefficients for the prespecified ordered model; its arrows indicate model specification and not temporal sequence.

**Table 4:** Total, direct, and indirect associations between exercise motivation and the ZTEx-IP^a^ involving general self-efficacy and perceived physical activity triggers (n=790)_b_.

Association component	B (SE)	95% CI
Total association	0.453 (0.034)	0.385-0.520
Direct association	0.167 (0.040)	0.088-0.246
Total indirect association	0.286 (0.034)	0.218-0.352
X → M1 → Y	0.104 (0.025)	0.057-0.155
X → M2 → Y	0.157 (0.024)	0.110-0.205
X → M1 → M2 → Y	0.025 (0.007)	0.013-0.039

^a^ZTEx-IP: zero-time exercise–related intention proxy.

^b^X: exercise motivation; Y: ZTEx-IP; M1: general self-efficacy; M2: perceived physical activity triggers; B: unstandardized coefficient. Indirect associations were estimated using 5000 bootstrap resamples and percentile 95% CIs. All equations were adjusted for age, income, incidental physical activity duration, structured exercise frequency, structured exercise duration, wearable-device use, and zero-time exercise familiarity.

### Theoretically Relevant Sensitivity Analyses

Analyses using the behavioral intention subscale or an outcome excluding perceived behavioral control items yielded positive direct and model-implied indirect associations, with the corresponding 95% CIs excluding 0. In the subgroup of participants who were at least somewhat familiar with ZTEx (n=187), the bootstrap CIs for both single-intermediate-variable associations and the total indirect association excluded 0, whereas the CIs for the direct and ordered indirect-association estimates included 0. An alternative variable ordering also yielded a nonzero ordered indirect-association estimate, reinforcing that the cross-sectional data could not identify a unique temporal order among the constructs. Complete numerical results and additional technical robustness analyses are reported in Multimedia Appendices 4 and 5.

## Discussion

### Principal Findings

In this study of 790 Chinese adults in sedentary occupations, higher exercise motivation was associated with higher ZTEx-IP scores. The association was graded and nonlinear across the observed motivation range. In both the parallel and ordered models, general self-efficacy and perceived physical activity triggers showed distinct model-implied indirect associations between exercise motivation and the ZTEx-IP. Perceived triggers had the larger specific indirect estimate and made the greater incremental contribution to explained variance, while the prespecified ordered term involving both intermediate variables yielded a smaller indirect estimate. Together, the adjusted variables explained 37.6% of the variance in the ZTEx-IP. The theoretically motivated sensitivity analyses retained the overall positive association pattern, although estimates in the ZTEx-familiar subgroup were less precise, and the alternative ordering confirmed that the cross-sectional data did not identify a unique temporal sequence. By considering these constructs in one model, the study extends prior work beyond isolated psychosocial correlates and highlights the joint relevance of motivational orientation, perceived agency, and cue-supportive contexts to readiness for brief, lifestyle-integrated movement.

### Interpretation, Comparison With Prior Work, and Implications

The graded association between exercise motivation and the ZTEx-IP is most plausibly interpreted as a relationship between a more self-determined regulatory profile and readiness to reorganize sedentary routines. The BREQ-2 assesses exercise regulation along an internalization continuum, capturing the quality and self-endorsement of motivation rather than the intensity of a momentary impulse [27,28]. This interpretation is consistent with evidence that perceived competence and more autonomous forms of regulation are recurrent correlates of physical activity [12]. The Chinese working-age context makes the finding particularly relevant. Half of Chinese adults aged 18-44 years report sitting for at least 6 hours per day [5], and longer sitting duration has been associated with a higher risk of major cardiovascular events among both manual and nonmanual workers [33]. For workers who find it difficult to set aside dedicated exercise time, an internalized value placed on movement may make brief bouts incorporated into existing routines more acceptable. The nonlinear pattern adds nuance but does not identify a threshold or plateau. Instead, it suggests that the strength of the motivation–ZTEx-IP association varies across the observed motivation range, providing a reason to test support tailored to workers’ motivational profiles without imposing arbitrary cutoffs. The 37.6% of variance explained by the adjusted model falls within the broad range reported for theory-based models of behavioral and physical activity intentions [34]. Direct comparison should nevertheless be cautious because the present outcome includes several Theory of Planned Behavior domains. The unexplained variance may reflect factors not captured in the model, including job control, workload, habit, time availability, organizational norms, and access to feasible opportunities for movement [35,36].

The model-implied indirect associations show that exercise motivation, generalized perceived agency, and recognizable movement opportunities covaried within the prespecified statistical model. General self-efficacy reflects confidence in handling challenges across situations rather than efficacy for a particular ZTEx task [30]. From an affordance perspective, workers reporting greater agency may also be more likely to regard a task transition, reminder, or scheduled break as a usable opportunity for movement [37]. This is one theoretically plausible interpretation, not evidence that self-efficacy temporally precedes trigger perception. The larger trigger-related estimate is also plausible for a low-threshold strategy such as ZTEx, which requires little time, space, equipment, or technical skill. A cue is useful only when movement is feasible within the task and acceptable in the workplace. This point is especially relevant in Chinese workplaces, where time pressure and limited social support have been reported as barriers to sustained activity, while locally tested programs increasingly combine digital delivery with managerial, team-based, or interactive support [38-41]. Perceived triggers may therefore reflect both cue exposure and the perceived legitimacy of responding to a cue. The co-occurrence of motivation, perceived agency, and salient opportunity is compatible with COM-B and the Fogg behavior model, but neither framework determines temporal order in these data. Trigger perception might enhance self-efficacy; both constructs might reflect unmeasured influences such as job control or workplace culture, or reciprocal relations might operate. The observed statistical pattern was consistent with the theory-informed ordering used in PROCESS Model 6; however, longitudinal or intensive repeated-measures research is required to determine whether and how these constructs develop over time.

The relative estimates identify perceived trigger exposure, exercise motivation, and general self-efficacy as constructs for hypothesis-driven intervention research rather than as intervention components ready for implementation. In the prespecified ordered model, the specific indirect estimate involving perceived triggers exceeded that involving general self-efficacy (B=0.157 vs B=0.104), and triggers made the larger incremental contribution to explained variance (Δ*R*²=0.054 vs 0.033). Because these observational estimates relate perceived triggers to the ZTEx-IP, they do not demonstrate that delivered prompts or changes in workplace practices affect actual ZTEx behavior. A primary hypothesis for prospective evaluation is that context-sensitive prompts may help translate intention into objectively measured sitting interruptions and ZTEx performance. Future trials could also examine whether any such effect varies according to baseline motivation, self-efficacy, or occupational context. Evidence that workplace prompts have modest and heterogeneous effects [42-44] and that the effects of team communication vary according to message type and frequency [38] underscores the need to evaluate, rather than assume, the added value of particular delivery or organizational components. The present data cannot determine which components, delivery modes, or combinations are effective. Future workplace trials should prespecify occupation-specific feasibility and safety criteria and assess objectively measured sitting interruptions, actual ZTEx frequency, adherence over time, and acceptability to workers and employers rather than intention alone [45].

### Limitations

This study had several strengths, including a relatively large and geographically diverse sample, standardized Chinese-language instruments with satisfactory internal consistency in the present sample, and extensive sensitivity analyses. Several limitations should nevertheless be considered. First, all variables were measured at one time point, so temporal precedence and causal relationships among exercise motivation, general self-efficacy, perceived physical activity triggers, and the ZTEx-IP could not be established. The model-implied indirect associations should therefore be understood as theory-informed decompositions of contemporaneous associations, not evidence of causal mediation. Reverse or bidirectional associations and alternative variable orderings remain possible [46-48]. Although the candidate variables were substantively motivated, the final adjustment set partly relied on nominal associations with the ZTEx-IP and was not derived from a prespecified causal graph. Outcome-based screening cannot identify confounding and may omit relevant variables with weak unadjusted associations. Residual confounding by workload, job control, workplace culture, or other excluded or unmeasured factors therefore remains possible. Second, the Adult Sedentary Behavior Reduction Intention Questionnaire has not been validated as a ZTEx-specific intention measure. It captures general willingness and perceived control regarding sedentary behavior reduction rather than intention to perform a specified ZTEx movement, and it does not directly assess ZTEx-specific knowledge, task feasibility, workplace appropriateness, or action planning [26]. This limitation is particularly important because 76.3% (603/790) of participants were unfamiliar with ZTEx and may have responded with reference to standing, walking, or other ways of interrupting sitting. Familiarity was measured with a single self-report item, and neither the standardized definition nor the sensitivity analyses could confirm ZTEx-specific understanding or construct validity. The ZTEx-IP should therefore be interpreted as a broad indicator of intention relevant to ZTEx adoption rather than a direct measure of ZTEx-specific intention. Future studies should develop and validate ZTEx-specific intention and readiness instruments. Third, although the instruments were reliable and theoretically relevant, they were not one-to-one operationalizations of COM-B or Fogg constructs. The BREQ-2 assesses the regulation and internalization of exercise motivation, the GSES assesses generalized perceived agency rather than confidence in performing ZTEx under specific work conditions, and the PATQ assesses perceived exposure to broad activity-related cues rather than prompts immediately preceding behavior [31]. Their differing behavioral and temporal referents further limit their correspondence with the proposed frameworks. Future research should use behavior-specific measures with aligned behavioral and temporal referents. Fourth, all principal variables were self-reported by the same participants at the same time. Common method variance, recall error, social desirability, and acquiescent responding may therefore have contributed to the observed associations. Although the first factor accounted for only 31.93% of the variance in Harman’s single-factor analysis, this diagnostic cannot exclude common method bias [49]. Temporally separated or objective assessments would provide stronger protection against this bias. Fifth, the study assessed an intention proxy rather than actual ZTEx uptake, the frequency of sitting interruptions, or behavioral maintenance. Higher ZTEx-IP scores therefore cannot be assumed to translate into ZTEx adoption or sustained practice [45]. Future studies should combine ZTEx-specific intention measures with objective sitting assessments, ZTEx performance records, and longitudinal follow-up. Finally, multichannel online convenience sampling did not provide a population-based sampling frame or a response-rate denominator, and the recruitment source was not recorded at the individual level. Individuals with greater interest in health, exercise, wearable devices, or digital health, as well as those more active in workplace WeChat groups or occupation-related online communities, may have been more likely to participate. This self-selection may have yielded more favorable psychosocial score profiles and may limit the transportability of the observed score distributions, reliability estimates, and associations. Individuals with limited digital access or less engagement with online workplace networks may also have been underrepresented. Generalizability is further limited by the inclusion of only 14 adults aged 60 years or older and by differences in occupational constraints on sitting interruption, particularly for drivers. Nonsignificant subgroup differences should not be interpreted as evidence of equivalence because some estimates were imprecise. Occupation-specific heterogeneity should be examined in adequately represented, independently recruited samples.

### Conclusions

Among Chinese adults in sedentary occupations, higher exercise motivation, general self-efficacy, and more frequent perceived physical activity triggers were jointly associated with higher scores on the ZTEx-IP. Perceived triggers had the largest specific indirect estimate and incremental contribution, but the findings do not establish a unique causal or temporal sequence or demonstrate that modifying these factors would increase actual ZTEx behavior. More broadly, intention relevant to ZTEx adoption may reflect the alignment of motivational orientation, perceived agency, and recognizable opportunities within everyday work routines rather than motivation alone. By integrating these theoretically distinct factors within an occupational context, this study identifies an empirically supported pattern that can inform the formulation and prioritization of hypotheses for prospective intervention research. Specifically, the findings support testing whether multicomponent strategies combining autonomy-supportive motivational content, graded movement options, context-sensitive prompts, and appropriate organizational support can facilitate the translation of intention into behavior. Workplace trials should determine whether these components affect objectively measured sitting interruptions, actual ZTEx frequency, adherence and maintenance over time, and workplace acceptability across desk-based and safety-sensitive occupations. Thus, rather than providing evidence of intervention effectiveness, the present study offers an empirically grounded and context-sensitive framework for advancing ZTEx research from intention-related associations to behavioral and intervention evaluation in Chinese occupational settings.

## Data Availability

The data analyzed in this study may be made available from the corresponding author upon reasonable request.

## Appendix

Multimedia Appendix 1 STROBE checklist.

Multimedia Appendix 2 Statistical methods and supplementary analyses.

Multimedia Appendix 3 Benjamini-Hochberg–adjusted results for group comparisons of the zero-time exercise–related intention proxy.

Multimedia Appendix 4 Regression diagnostics and technical robustness analyses for the adjusted regression models.

Multimedia Appendix 5 Theoretically motivated sensitivity analyses and secondary robustness checks for the indirect-association models.

Multimedia Appendix 6 Multivariable-adjusted associations between exercise motivation tertiles and the zero-time exercise–related intention proxy among Chinese adults in sedentary occupations.

Multimedia Appendix 7 Factor loadings from Harman’s single-factor test using all core questionnaire items.

Multimedia Appendix 8 Adjusted parallel indirect-association model (PROCESS Model 4) results.

## Abbreviations

BootSE: bootstrap SE
BREQ-2: Behavioral Regulation in Exercise Questionnaire
COM-B: capability, opportunity, motivation-behavior
GSES: General Self-Efficacy Scale
MET: metabolic equivalent
PATQ: Physical Activity Triggers Questionnaire
STROBE: Strengthening the Reporting of Observational Studies in Epidemiology
ZTEx: zero-time exercise
ZTEx-IP: zero-time exercise–related intention proxy
