# Supplementary material for: Psychosocial Correlates of Sedentary Behavior Reduction Readiness Relevant to Zero-Time Exercise Among Chinese Adults in Sedentary Occupations: Cross-Sectional Study
Source: JMIR Public Health Surveill. 2026 Sep 4;12:e95707. doi: 10.2196/95707 (PMC13589206; doi:10.2196/95707)
Supplement: Supplementary file 2 [file publichealth_v12i1e95707_app2.docx]

**Multimedia Appendix 1. Statistical Methods and Supplementary Analyses**

1. **Scenario-Based Monte Carlo Sample-Adequacy Analysis for the Ordered Indirect-Association Model**

This supplementary analysis evaluated the sample size available to detect the specific three-path ordered indirect association in the prespecified model: exercise motivation → general self-efficacy → perceived physical activity triggers → ZTEx-related intention proxy (ZTEx-IP).

A model-specific Monte Carlo simulation was used because general sample-size recommendations for simple indirect-association models do not directly quantify power for a three-path ordered indirect association.

All focal variables were represented as standardized continuous variables. The simulated population model included the paths X → M1 (a₁), X → M2 (a₂), M1 → M2 (d₂₁), X → Y (c′), M1 → Y (b₁), and M2 → Y (b₂). Residual variances were selected so that each simulated endogenous variable had a variance of approximately 1.

For each condition, 5,000 independent datasets were generated and the ordered indirect-association model was fitted using maximum likelihood estimation in lavaan. Statistical significance was evaluated at a two-sided alpha level of .05. Power was defined as the proportion of simulated 95% confidence intervals for a₁d₂₁b₂ that excluded zero. Confidence intervals for a₁d₂₁b₂ were calculated using the first-order delta method.

Simulation precision was summarized using the Monte Carlo standard error, calculated as √[p(1-p)/R], where p is the estimated power and R is the number of valid simulated datasets. Approximate 95% Monte Carlo uncertainty intervals were calculated as p ± 1.96 × Monte Carlo standard error.

Two hypothetical scenarios were evaluated:

**Hypothetical small-to-moderate scenario:** The constituent paths were set to a₁=0.26, d₂₁=0.14, and b₂=0.26, producing an expected ordered indirect association of 0.009464. The remaining focal paths were set to a₂=0.26, b₁=0.26, and c′=0.20.

**Conservative very-small-association scenario:** The constituent paths were set to a₁=0.20, d₂₁=0.10, and b₂=0.20, producing an expected ordered indirect association of 0.0040. The remaining focal paths were set to a₂=0.20, b₁=0.20, and c′=0.20.

At N=790, all 5,000 simulated models converged in both scenarios. Table 1 summarizes the estimated power, Monte Carlo uncertainty, and convergence results.

**Table S1.** Monte Carlo power for the three-path ordered indirect association at N=790

| **Scenario** | **Constituent paths (a₁, d₂₁, b₂)** | **Ordered indirect association** | **N** | **Estimated power** | **Approx. 95% Monte Carlo interval** | **Convergence** |
| --- | --- | --- | --- | --- | --- | --- |
| Conservative very-small association | 0.20, 0.10, 0.20 | 0.0040 | 790 | 70.6% | 69.4%-71.9% | 100% |
| Hypothetical small-to-moderate association | 0.26, 0.14, 0.26 | 0.0095 | 790 | 97.0% | 96.6%-97.5% | 100% |

**Note.** Power was defined as the proportion of simulated 95% confidence intervals for a₁d₂₁b₂ that excluded zero. Each condition included 5,000 simulated datasets. The Monte Carlo intervals quantify simulation uncertainty in the estimated power and are not confidence intervals for the ordered indirect association itself.

The hypothetical small-to-moderate scenario was additionally evaluated across sample sizes of 200, 250, 300, 325, 400, 500, 600, 650, 700, 790, 900, 1,000, and 1,200. Within this examined grid, approximately 500 participants were required to achieve at least 80% power, and approximately 600 participants were required to achieve at least 90% power. At N=790, estimated power was 97.0%.

**Figure S1.** Monte Carlo power curve for the three-path ordered indirect association. The simulation assumed standardized constituent paths of a₁=0.26, d₂₁=0.14, and b₂=0.26, corresponding to an expected ordered indirect association of 0.0095. Each sample-size condition included 5,000 simulated datasets.

At N=790, estimated power was 97.0% under the hypothetical small-to-moderate scenario and 70.6% under the conservative very-small-association scenario. The study may therefore have been less sensitive to substantially weaker ordered indirect associations.
