# Supplementary material for: Psychosocial Correlates of Sedentary Behavior Reduction Readiness Relevant to Zero-Time Exercise Among Chinese Adults in Sedentary Occupations: Cross-Sectional Study
Source: JMIR Public Health Surveill. 2026 Sep 4;12:e95707. doi: 10.2196/95707 (PMC13589206; doi:10.2196/95707)
Supplement: Supplementary file 3 [file publichealth_v12i1e95707_app3.docx]

**Multimedia Appendix 3. Benjamini-Hochberg-Adjusted Results for Group Comparisons of the ZTEx-IP.**

| Variable | Statistical test | Statistic | Raw P value | BH-adjusted P value |
| --- | --- | --- | --- | --- |
| Gender | Mann-Whitney U test | U=75860.5 | .801 | .801 |
| Age | Kruskal-Wallis H test | H=12.197 | .016 | .040 |
| Educational attainment | Kruskal-Wallis H test | H=2.086 | .352 | .378 |
| Average monthly income (RMB) | Kruskal-Wallis H test | H=8.631 | .035 | .074 |
| Occupation category | Kruskal-Wallis H test | H=6.881 | .076 | .142 |
| Job tenure (years) | Kruskal-Wallis H test | H=5.802 | .122 | .203 |
| Cumulative daily sedentary duration (hours) | Kruskal-Wallis H test | H=3.524 | .318 | .367 |
| Musculoskeletal discomfort | Kruskal-Wallis H test | H=3.669 | .300 | .367 |
| Health examination within the past 6 months | Mann-Whitney U test | U=79216.0 | .237 | .324 |
| Chronic disease | Mann-Whitney U test | U=66102.0 | .169 | .254 |
| Cumulative duration of daily incidental physical activity (min) | Kruskal-Wallis H test | H=18.938 | <.001 | .001 |
| Frequency of *structured exercise* (sessions/week) | Kruskal-Wallis H test | H=69.460 | <.001 | <.001 |
| Average duration per structured exercise session (min) | Kruskal-Wallis H test | H=61.993 | <.001 | <.001 |
| Use of wearable health-tracking devices | Mann-Whitney U test | U=80325.0 | <.001 | .001 |
| Familiarity with ZTEx | Kruskal-Wallis H test | H=8.457 | .015 | .040 |
