# Supplementary material for: Psychosocial Correlates of Sedentary Behavior Reduction Readiness Relevant to Zero-Time Exercise Among Chinese Adults in Sedentary Occupations: Cross-Sectional Study
Source: JMIR Public Health Surveill. 2026 Sep 4;12:e95707. doi: 10.2196/95707 (PMC13589206; doi:10.2196/95707)
Supplement: Supplementary file 4 [file publichealth_v12i1e95707_app4.docx]

**Multimedia Appendix 4. Regression Diagnostics and Technical Robustness Analyses for the Adjusted Regression Models**

This appendix reports regression diagnostics and secondary technical robustness checks for the 3 adjusted component equations of the prespecified ordered indirect-association model. Diagnostics included multicollinearity, residual distribution, heteroscedasticity, and influential observations. HC3 heteroscedasticity-consistent standard errors and categorical specifications of age and income were examined as robustness checks.

**Table 1.** Variance Inflation Factor Values for the Multivariable Model

| Predictor | VIF |
| --- | --- |
| Exercise motivation | 1.885 |
| General self-efficacy | 1.420 |
| Perceived physical activity triggers | 1.772 |
| Age | 1.043 |
| Income | 1.061 |
| Incidental physical activity duration | 1.128 |
| Structured exercise frequency | 1.879 |
| Structured exercise duration | 1.834 |
| Wearable device use | 1.064 |
| ZTEx familiarity | 1.107 |

**Note.** VIF = variance inflation factor; ZTEx = zero-time exercise. The multivariable model included exercise motivation, general self-efficacy, perceived physical activity triggers, age, income, incidental physical activity duration, structured exercise frequency, structured exercise duration, wearable-device use, and ZTEx familiarity. All VIF values were below 2.0, indicating no evidence of problematic multicollinearity.

**Figure 1.** Regression diagnostic plots for the general self-efficacy equation in the ordered indirect-association model (PROCESS Model 6). Diagnostic plots for the regression equation predicting general self-efficacy from exercise motivation and the covariates. The plots include residual-versus-fitted values, normal Q-Q residuals, scale-location, and residual-versus-leverage.

**Figure 2.** Regression diagnostic plots for the perceived physical activity triggers equation in the ordered indirect-association model (PROCESS Model 6). Diagnostic plots for the regression equation predicting perceived physical activity triggers from exercise motivation, general self-efficacy, and the covariates. The plots include residual-versus-fitted values, normal Q-Q residuals, scale-location, and residual-versus-leverage.

**Figure 3.** Regression diagnostic plots for the ZTEx-related intention proxy equation in the ordered indirect-association model (PROCESS Model 6). Diagnostic plots for the regression equation predicting the ZTEx-related intention proxy from exercise motivation, general self-efficacy, perceived physical activity triggers, and the covariates. The plots include residual-versus-fitted values, normal Q-Q residuals, scale-location, and residual-versus-leverage.

**Table 2.** HC3 robust standard error results for the three regression equations in the prespecified ordered indirect-association model.

| Outcome | Predictor | B | Robust SE | 95% CI | P |
| --- | --- | --- | --- | --- | --- |
| General self-efficacy | Exercise motivation | 0.214 | 0.021 | 0.173 to 0.255 | <.001 |
| General self-efficacy | Age | 0.067 | 0.160 | -0.246 to 0.380 | .675 |
| General self-efficacy | Income | 0.537 | 0.233 | 0.079 to 0.994 | .022 |
| General self-efficacy | Incidental physical activity duration | 0.236 | 0.188 | -0.133 to 0.605 | .209 |
| General self-efficacy | Structured exercise frequency | 0.773 | 0.294 | 0.196 to 1.349 | .009 |
| General self-efficacy | Structured exercise duration | -0.036 | 0.262 | -0.549 to 0.478 | .892 |
| General self-efficacy | Wearable device use | 0.160 | 0.364 | -0.556 to 0.875 | .661 |
| General self-efficacy | ZTEx familiarity | -0.218 | 0.350 | -0.906 to 0.469 | .534 |
| Perceived physical activity triggers | Exercise motivation | 0.504 | 0.041 | 0.423 to 0.584 | <.001 |
| Perceived physical activity triggers | General self-efficacy | 0.378 | 0.080 | 0.221 to 0.535 | <.001 |
| Perceived physical activity triggers | Age | -0.707 | 0.355 | -1.403 to -0.012 | .046 |
| Perceived physical activity triggers | Income | -0.359 | 0.424 | -1.191 to 0.473 | .397 |
| Perceived physical activity triggers | Incidental physical activity duration | 0.098 | 0.358 | -0.605 to 0.801 | .784 |
| Perceived physical activity triggers | Structured exercise frequency | 0.492 | 0.577 | -0.640 to 1.624 | .394 |
| Perceived physical activity triggers | Structured exercise duration | -0.176 | 0.516 | -1.188 to 0.836 | .733 |
| Perceived physical activity triggers | Wearable device use | -2.507 | 0.710 | -3.901 to -1.114 | <.001 |
| Perceived physical activity triggers | ZTEx familiarity | 2.831 | 0.706 | 1.444 to 4.218 | <.001 |
| ZTEx-related intention proxy | Exercise motivation | 0.167 | 0.051 | 0.066 to 0.268 | .001 |
| ZTEx-related intention proxy | General self-efficacy | 0.483 | 0.103 | 0.280 to 0.686 | <.001 |
| ZTEx-related intention proxy | Perceived physical activity triggers | 0.311 | 0.047 | 0.219 to 0.404 | <.001 |
| ZTEx-related intention proxy | Age | 0.623 | 0.360 | -0.084 to 1.331 | .084 |
| ZTEx-related intention proxy | Income | 0.492 | 0.485 | -0.459 to 1.444 | .310 |
| ZTEx-related intention proxy | Incidental physical activity duration | 0.344 | 0.389 | -0.420 to 1.108 | .377 |
| ZTEx-related intention proxy | Structured exercise frequency | 0.799 | 0.603 | -0.384 to 1.983 | .185 |
| ZTEx-related intention proxy | Structured exercise duration | 0.733 | 0.533 | -0.313 to 1.779 | .169 |
| ZTEx-related intention proxy | Wearable device use | -0.591 | 0.724 | -2.013 to 0.831 | .415 |
| ZTEx-related intention proxy | ZTEx familiarity | -1.211 | 0.681 | -2.548 to 0.126 | .076 |

**Note.** HC3 heteroscedasticity-consistent standard errors were calculated separately for all three regression equations in the prespecified ordered indirect-association model. B values are unstandardized coefficients. The models were adjusted for age, income, incidental physical activity duration, structured exercise frequency, structured exercise duration, wearable-device use, and ZTEx familiarity. ZTEx=zero-time exercise; CI=confidence interval.

**Table 3.** Comparison of linear and categorical specifications of age and income across adjusted regression equations.

| Equation | Specification comparison | df difference | F value | P value |
| --- | --- | --- | --- | --- |
| Adjusted total-association model | Age: linear vs categorical | 3 | 4.213 | 0.006 |
| Adjusted total-association model | Income: linear vs categorical | 2 | 0.286 | 0.752 |
| Adjusted total-association model | Age and income: joint comparison | 5 | 2.639 | 0.022 |
| General self-efficacy equation | Age: linear vs categorical | 3 | 0.555 | 0.645 |
| General self-efficacy equation | Income: linear vs categorical | 2 | 0.655 | 0.520 |
| General self-efficacy equation | Age and income: joint comparison | 5 | 0.615 | 0.688 |
| Trigger equation: parallel model | Age: linear vs categorical | 3 | 0.475 | 0.700 |
| Trigger equation: parallel model | Income: linear vs categorical | 2 | 1.558 | 0.211 |
| Trigger equation: parallel model | Age and income: joint comparison | 5 | 0.957 | 0.444 |
| Trigger equation: ordered model | Age: linear vs categorical | 3 | 0.609 | 0.609 |
| Trigger equation: ordered model | Income: linear vs categorical | 2 | 1.901 | 0.150 |
| Trigger equation: ordered model | Age and income: joint comparison | 5 | 1.187 | 0.313 |
| Intention-proxy outcome equation | Age: linear vs categorical | 3 | 4.954 | 0.002 |
| Intention-proxy outcome equation | Income: linear vs categorical | 2 | 0.742 | 0.477 |
| Intention-proxy outcome equation | Age and income: joint comparison | 5 | 3.270 | 0.006 |

**Note.** F tests compared adjusted models specifying age and/or income as linear covariates with corresponding models specifying these covariates as categorical variables. Other covariates were kept unchanged across comparisons.

**Table 4.** Sensitivity of exercise motivation coefficients to alternative covariate specifications.

| Equation | B for exercise motivation: linear covariates | B for exercise motivation: flexible covariates | Change in B | Percent change, % |
| --- | --- | --- | --- | --- |
| Adjusted total-association model | 0.453 | 0.454 | 0.001 | 0.28 |
| General self-efficacy equation | 0.214 | 0.215 | 0.000 | 0.21 |
| Trigger equation: parallel model | 0.584 | 0.584 | -0.000 | -0.08 |
| Trigger equation: ordered model | 0.504 | 0.501 | -0.002 | -0.42 |
| Intention-proxy outcome equation | 0.167 | 0.167 | -0.001 | -0.33 |

**Note.** Linear covariate models treated age and income as continuous/ordinal linear covariates, whereas flexible covariate models treated age and income as categorical variables. Change in B was calculated as the coefficient from the flexible model minus the coefficient from the linear covariate model. The very small changes indicate that the estimated associations involving exercise motivation were stable under alternative age and income specifications.
