# Supplementary material for: Psychosocial Correlates of Sedentary Behavior Reduction Readiness Relevant to Zero-Time Exercise Among Chinese Adults in Sedentary Occupations: Cross-Sectional Study
Source: JMIR Public Health Surveill. 2026 Sep 4;12:e95707. doi: 10.2196/95707 (PMC13589206; doi:10.2196/95707)
Supplement: Supplementary file 5 [file publichealth_v12i1e95707_app5.docx]

**Multimedia Appendix 5. Theoretically Motivated Sensitivity Analyses and Secondary Robustness Checks for the Indirect-Association Models**

1. **Theoretically Motivated Sensitivity Analyses: Alternative Outcome Definitions and ZTEx Familiarity Restriction**

Three additional sensitivity analyses were conducted to assess the robustness of the principal findings. Sensitivity analysis 1 replaced the outcome with the behavioral intention subscale (AS1, AS5, and AS11). Sensitivity analysis 2 excluded the perceived behavioral control items (AS4 and AS8) from the outcome measure. Sensitivity analysis 3 restricted the analyses to participants who reported being at least somewhat familiar with zero-time exercise (ZTEx). For each analysis, the indirect-association analyses were repeated using PROCESS Model 4 (parallel specification) and Model 6 (ordered specification), while all other model specifications remained unchanged unless otherwise stated.

**Table 1.** Sensitivity analysis 1: Using the behavioral intention subscale (AS1, AS5, and AS11) as the outcome

A. PROCESS Model 4

| **Association component** | **Main analysis** | **Sensitivity analysis** |
| --- | --- | --- |
| Direct association | 0.167 (95% CI 0.088-0.246), P<.001 | 0.0119 (95% CI 0.0059-0.0179), P=.0001 |
| Indirect association involving general self-efficacy | 0.104 (95% CI 0.057-0.155) | 0.0068 (95% CI 0.0034-0.0106) |
| Indirect association involving perceived physical activity triggers | 0.182 (95% CI 0.129-0.233) | 0.0121 (95% CI 0.0082-0.0159) |
| Total indirect association | 0.286 (95% CI 0.220-0.353) | 0.0189 (95% CI 0.0141-0.0236) |
| Model R² | 0.376 | 0.321 |

B. PROCESS Model 6

| **Association component** | **Main analysis** | **Sensitivity analysis** |
| --- | --- | --- |
| Direct association | 0.167 (95% CI 0.088-0.246), P<.001 | 0.0119 (95% CI 0.0059-0.0179), P=.0001 |
| Indirect association involving general self-efficacy | 0.104 (95% CI 0.057-0.155) | 0.0068 (95% CI 0.0033-0.0107) |
| Indirect association involving perceived physical activity triggers | 0.157 (95% CI 0.110-0.205) | 0.0104 (95% CI 0.0069-0.0141) |
| Ordered indirect association involving both intermediate variables | 0.025 (95% CI 0.013-0.039) | 0.0017 (95% CI 0.0009-0.0026) |
| Total indirect association | 0.286 (95% CI 0.218-0.352) | 0.0189 (95% CI 0.0140-0.0238) |
| Model R² | 0.376 | 0.321 |

**Note.** Outcome variable was replaced with the behavioral intention subscale (AS1, AS5, and AS11). All covariates and analytical procedures were identical to those used in the primary analyses.

**Table 2.** Sensitivity analysis 2: Excluding perceived behavioral control items (AS4 and AS8) from the outcome

A. PROCESS Model 4

| **Association component** | **Main analysis** | **Sensitivity analysis** |
| --- | --- | --- |
| Direct association | 0.167 (95% CI 0.088-0.246), P<.001 | 0.1483 (95% CI 0.0796-0.2170), P<.001 |
| Indirect association involving general self-efficacy | 0.104 (95% CI 0.057-0.155) | 0.0859 (95% CI 0.0465-0.1308) |
| Indirect association involving perceived physical activity triggers | 0.182 (95% CI 0.129-0.233) | 0.1594 (95% CI 0.1119-0.2031) |
| Total indirect association | 0.286 (95% CI 0.220-0.353) | 0.2453 (95% CI 0.1879-0.3015) |
| Model R² | 0.376 | 0.374 |

B. PROCESS Model 6

| **Association component** | **Main analysis** | **Sensitivity analysis** |
| --- | --- | --- |
| Direct association | 0.167 (95% CI 0.088-0.246), P<.001 | 0.1483 (95% CI 0.0796-0.2170), P<.001 |
| Indirect association involving general self-efficacy | 0.104 (95% CI 0.057-0.155) | 0.0859 (95% CI 0.0449-0.1320) |
| Indirect association involving perceived physical activity triggers | 0.157 (95% CI 0.110-0.205) | 0.1373 (95% CI 0.0968-0.1781) |
| Ordered indirect association involving both intermediate variables | 0.025 (95% CI 0.013-0.039) | 0.0221 (95% CI 0.0118-0.0342) |
| Total indirect association | 0.286 (95% CI 0.218-0.352) | 0.2453 (95% CI 0.1888-0.3036) |
| Model R² | 0.376 | 0.374 |

**Note.** The outcome was recalculated after excluding the perceived behavioral control items (AS4 and AS8). All remaining analytical procedures were unchanged.

**Table 3.** Sensitivity analysis 3: Restricting the analysis to participants who were at least somewhat familiar with ZTEx (n = 187)

A. PROCESS Model 4

| **Association component** | **Main analysis** | **Restricted sample** |
| --- | --- | --- |
| Direct association | 0.167 (95% CI 0.088-0.246), P<.001 | 0.0531 (95% CI -0.0916 to 0.1978), P=.470 |
| Indirect association involving general self-efficacy | 0.104 (95% CI 0.057-0.155) | 0.1189 (95% CI 0.0435-0.1973) |
| Indirect association involving perceived physical activity triggers | 0.182 (95% CI 0.129-0.233) | 0.2075 (95% CI 0.1012-0.3309) |
| Total indirect association | 0.286 (95% CI 0.220-0.353) | 0.3264 (95% CI 0.1960-0.4682) |
| Model R² | 0.376 | 0.461 |

B. PROCESS Model 6

| **Association component** | **Main analysis** | **Restricted sample** |
| --- | --- | --- |
| Direct association | 0.167 (95% CI 0.088-0.246), P<.001 | 0.0531 (95% CI -0.0916 to 0.1978), P=.470 |
| Indirect association involving general self-efficacy | 0.104 (95% CI 0.057-0.155) | 0.1189 (95% CI 0.0441-0.2010) |
| Indirect association involving perceived physical activity triggers | 0.157 (95% CI 0.110-0.205) | 0.1775 (95% CI 0.0809-0.3004) |
| Ordered indirect association involving both intermediate variables | 0.025 (95% CI 0.013-0.039) | 0.0300 (95% CI -0.0073 to 0.0717) |
| Total indirect association | 0.286 (95% CI 0.218-0.352) | 0.3264 (95% CI 0.1968-0.4655) |
| Model R² | 0.376 | 0.461 |

**Note.** Analyses were repeated after restricting the sample to participants who reported being at least somewhat familiar with ZTEx. Because the sample size decreased substantially (790 to 187), wider bootstrap confidence intervals were expected.

2. **Alternative Ordering of the Intermediate Variables**

To examine whether the findings depended on the ordering of the intermediate variables, we compared 2 simplified ordered path models using structural equation modeling. The prespecified model used the ordering:

motivation → self-efficacy → perceived physical activity triggers → ZTEx-related intention proxy (ZTEx-IP)

The alternative-ordering model used:

perceived physical activity triggers → motivation → self-efficacy → ZTEx-related intention proxy (ZTEx-IP)

Motivation, general self-efficacy, perceived physical activity triggers, and the ZTEx-related intention proxy (ZTEx-IP) were entered as observed composite scores. Both models were estimated using robust maximum likelihood estimation with Yuan-Bentler scaled test statistics. The exogenous variable was allowed to covary with the adjustment variables.

Ordered indirect, direct, and total associations were additionally estimated using 5000 percentile bootstrap samples. These simplified path models were used only to compare alternative variable orderings and were not intended to replace the prespecified adjusted regression models.

**Table 4.** Model-fit comparison between the prespecified and alternative-ordering models

| Model | Robust χ² (df) | P value | Robust CFI | Robust TLI | Robust RMSEA (90% CI) | SRMR | AIC | BIC |
| --- | --- | --- | --- | --- | --- | --- | --- | --- |
| Prespecified-ordering model: Motivation → self-efficacy → perceived triggers → intention | 142.54 (2) | <.001 | 0.849 | 0.548 | 0.304 (0.263-0.348) | 0.098 | 22698.14 | 22735.52 |
| Alternative-ordering model: Perceived triggers → motivation → self-efficacy → intention | 38.46 (2) | <.001 | 0.952 | 0.856 | 0.172 (0.127-0.221) | 0.055 | 22598.82 | 22636.19 |

**Note.** N=790. Models were estimated using robust maximum likelihood estimation. CFI=comparative fit index; TLI=Tucker-Lewis index; RMSEA=root mean square error of approximation; SRMR=standardized root mean square residual; AIC=Akaike information criterion; BIC=Bayesian information criterion. The AIC and BIC values of the alternative-ordering model were each 99.32 points lower than those of the prespecified-ordering model.

**Table 5.** Path coefficients in the prespecified and alternative-ordering models

| **Model** | **Path** | **B** | **SE** | **95% CI** | **Standardized β** | **P value** |
| --- | --- | --- | --- | --- | --- | --- |
| Prespecified ordering | Motivation → self-efficacy | 0.641 | 0.032 | 0.578-0.704 | 0.620 | <.001 |
| Prespecified ordering | Self-efficacy → perceived triggers | 0.195 | 0.017 | 0.163-0.227 | 0.433 | <.001 |
| Prespecified ordering | Perceived triggers → intention | 0.657 | 0.105 | 0.452-0.862 | 0.302 | <.001 |
| Prespecified ordering | Motivation → intention | 0.353 | 0.045 | 0.266-0.441 | 0.348 | <.001 |
| Alternative ordering | Perceived triggers → motivation | 1.060 | 0.090 | 0.884-1.235 | 0.493 | <.001 |
| Alternative ordering | Motivation → self-efficacy | 0.641 | 0.032 | 0.578-0.704 | 0.620 | <.001 |
| Alternative ordering | Self-efficacy → intention | 0.393 | 0.037 | 0.321-0.465 | 0.398 | <.001 |
| Alternative ordering | Perceived triggers → intention | 0.653 | 0.096 | 0.464-0.841 | 0.297 | <.001 |

**Note.** B represents the unstandardized coefficient. SEs and P values were obtained using robust maximum likelihood estimation. “Intention” refers to the ZTEx-related intention proxy (ZTEx-IP) used as the outcome in the primary analysis.

**Table 6.** Bootstrap indirect, direct, and total associations

| **Model** | **Association component** | **B** | **BootSE** | **Bootstrap 95% CI** | **Standardized association** | **P value** |
| --- | --- | --- | --- | --- | --- | --- |
| Prespecified ordering | Ordered indirect association: motivation → self-efficacy → perceived triggers → intention | 0.082 | 0.017 | 0.051-0.117 | 0.081 | <.001 |
|  | Direct association: motivation → intention | 0.353 | 0.045 | 0.264-0.442 | 0.348 | <.001 |
|  | Total association | 0.435 | 0.041 | 0.352-0.512 | 0.429 | <.001 |
| Alternative ordering | Ordered indirect association: perceived triggers → motivation → self-efficacy → intention | 0.267 | 0.037 | 0.196-0.341 | 0.122 | <.001 |
|  | Direct association: perceived triggers → intention | 0.653 | 0.096 | 0.461-0.839 | 0.297 | <.001 |
|  | Total association | 0.920 | 0.098 | 0.721-1.103 | 0.419 | <.001 |

**Note.** Confidence intervals were estimated using 5000 percentile bootstrap samples. BootSE=bootstrap standard error. Because the models have different starting variables and the variables use different score ranges, the unstandardized direct and total associations should not be compared as though they were on the same scale. The standardized ordered indirect associations are more suitable for descriptive comparison.

3. **Secondary Robustness Check 1: Influence Diagnostics for an Alternative Ordering**

Influence diagnostics were conducted for an alternative ordered model in which perceived physical activity triggers preceded general self-efficacy: exercise motivation → perceived physical activity triggers → general self-efficacy → ZTEx-IP.

Cook’s distance was calculated separately for the 3 regression equations constituting this alternative model: exercise motivation predicting perceived physical activity triggers; exercise motivation and perceived physical activity triggers predicting general self-efficacy; and exercise motivation, perceived physical activity triggers, and general self-efficacy predicting the ZTEx-related intention proxy (ZTEx-IP). A conservative threshold of Cook’s D greater than 4/N was used, corresponding to 0.00506 for the full sample of 790 participants. An observation was classified as influential if it exceeded this threshold in at least 1 equation.

After observations classified as influential were excluded, the alternative ordered model was re-estimated using the same outcome, covariate set, and variable ordering. HC3 heteroscedasticity-consistent standard errors were used for the component regressions, and indirect associations were estimated using 5000 percentile bootstrap samples.

**Table 7.** Influential observations identified in the alternative ordered model using Cook’s distance

| Regression equation | Observations exceeding Cook’s D > 4/N, n | Percentage |
| --- | --- | --- |
| Self-efficacy equation | 41 | 5.19% |
| Perceived physical activity triggers equation | 60 | 7.59% |
| ZTEx-related intention proxy (ZTEx-IP) equation | 49 | 6.20% |
| At least 1 equation | 114 | 14.43% |

**Note.** N=790. The Cook’s distance threshold was 4/N=0.00506. An observation was classified as influential when it exceeded the threshold in at least 1 regression equation. Because some observations exceeded the threshold in more than 1 equation, the equation-specific counts do not sum to the number of unique influential observations.

**Table 8.** Core model coefficients in the alternative ordered model before and after excluding influential observations

| **Path** | **Full sample B** | **HC3 SE** | **P value** | **Reduced sample B** | **HC3 SE** | **P value** |
| --- | --- | --- | --- | --- | --- | --- |
| Motivation → perceived triggers | 0.584 | 0.036 | <.001 | 0.659 | 0.031 | <.001 |
| Motivation → self-efficacy | 0.158 | 0.024 | <.001 | 0.105 | 0.019 | <.001 |
| Perceived triggers → self-efficacy | 0.096 | 0.019 | <.001 | 0.093 | 0.019 | <.001 |
| Perceived triggers → intention | 0.311 | 0.047 | <.001 | 0.163 | 0.039 | <.001 |
| Self-efficacy → intention | 0.483 | 0.103 | <.001 | 0.577 | 0.096 | <.001 |
| Motivation → intention | 0.167 | 0.051 | .001 | 0.186 | 0.041 | <.001 |

**Note.** Full sample, N=790; reduced sample after excluding influential observations, n=676. HC3 heteroscedasticity-consistent standard errors were used. “Intention” refers to the ZTEx-related intention proxy (ZTEx-IP). This alternative specification placed perceived physical activity triggers before general self-efficacy and differed from the primary PROCESS Model 6.

**Table 9.** Direct and model-implied indirect associations in the alternative ordered model before and after excluding influential observations

| **Association component** | **Full sample, B** | **BootSE** | **Bootstrap 95% CI** | **Reduced sample, B** | **BootSE** | **Bootstrap 95% CI** | **Change** |
| --- | --- | --- | --- | --- | --- | --- | --- |
| Direct association: motivation → intention | 0.167 | 0.050 | 0.071-0.268 | 0.186 | 0.041 | 0.107-0.267 | +11.42% |
| Motivation → perceived triggers → intention | 0.182 | 0.026 | 0.129-0.233 | 0.107 | 0.026 | 0.058-0.158 | -40.96% |
| Motivation → self-efficacy → intention | 0.076 | 0.020 | 0.040-0.120 | 0.060 | 0.015 | 0.033-0.094 | -21.01% |
| Motivation → perceived triggers → self-efficacy → intention | 0.027 | 0.008 | 0.013-0.045 | 0.035 | 0.010 | 0.018-0.055 | +31.45% |
| Total indirect association | 0.286 | 0.033 | 0.220-0.349 | 0.203 | 0.030 | 0.146-0.262 | -28.77% |
| Total association | 0.453 | 0.043 | 0.366-0.535 | 0.390 | 0.032 | 0.326-0.454 | -13.92% |

**Note.** Full sample, N=790; reduced sample after excluding 114 influential observations, n=676. Confidence intervals were estimated using 5000 nonparametric percentile bootstrap samples. All reported bootstrap confidence intervals excluded 0. Percentage change was calculated as 100 × (reduced-sample estimate − full-sample estimate) / |full-sample estimate|. Because this analysis used an alternative ordering of the intermediate variables, its estimates should be interpreted as evidence concerning specification sensitivity rather than as a numerical replication of the primary PROCESS Model 6.

4. **Secondary Robustness Check 2: Alternative Estimation Using Structural Equation Modeling**

As a secondary technical robustness check, the prespecified ordered indirect-association model was re-estimated using structural equation modeling (SEM) in lavaan. The predictor, intermediate variables, ordering, outcome, covariates, and 5000-bootstrap procedure were retained; robust maximum likelihood estimation was used.

**Table 10.** Indirect and Total Associations Estimated Using Structural Equation Modeling

| Association component | B | BootSE | Bootstrap 95% CI | Standardized association | P value |
| --- | --- | --- | --- | --- | --- |
| Motivation → general self-efficacy → intention | 0.103 | 0.026 | 0.056-0.156 | 0.100 | <.001 |
| Motivation → perceived physical activity triggers → intention | 0.157 | 0.024 | 0.109-0.205 | 0.151 | <.001 |
| Motivation → general self-efficacy → perceived physical activity triggers → intention | 0.025 | 0.006 | 0.013-0.039 | 0.024 | <.001 |
| Total indirect association | 0.286 | 0.034 | 0.218-0.354 | 0.275 | <.001 |
| Total association | 0.453 | 0.043 | 0.365-0.535 | 0.436 | <.001 |

**Note.** N=790. Indirect associations were estimated using structural equation modeling with 5000 percentile bootstrap samples. BootSE=bootstrap standard error. The SEM point estimates agreed with PROCESS Model 6 to 3 decimal places because the same observed-variable equations were re-estimated; this result is an estimation check rather than independent validation.

5. **Secondary Robustness Check 3: Nonlinear Specification of the Ordered Indirect-Association Model**

Because the prespecified ordered indirect-association model assumed linear associations, whereas restricted cubic spline analyses indicated nonlinearity between exercise motivation and the 2 intermediate variables and the ZTEx-IP, a secondary nonlinear specification was examined. General self-efficacy and perceived physical activity triggers were modeled as restricted cubic spline functions of exercise motivation, while their associations with the ZTEx-IP remained linear.

Restricted cubic spline specifications had lower AIC values than the corresponding linear specifications for all 3 component equations (ΔAIC, −7.64 to −29.74). Interval-specific direct and model-implied indirect associations were then estimated across the observed motivation range; results are summarized in Table 12.

**Table 11.** Comparison of Linear and Restricted Cubic Spline Specifications for the Three Component Equations

| Regression equation | Overall P value | P for nonlinearity | Linear AIC | RCS AIC | ΔAIC |
| --- | --- | --- | --- | --- | --- |
| General self-efficacy equation | <.001 | <.001 | 4716.58 | 4695.23 | -21.35 |
| Perceived physical activity triggers equation | <.001 | .003 | 5775.27 | 5767.63 | -7.64 |
| ZTEx-related intention proxy (ZTEx-IP) equation | <.001 | <.001 | 5861.96 | 5832.23 | -29.74 |

**Note.** N=790. RCS=restricted cubic spline; AIC=Akaike information criterion; ΔAIC=RCS AIC minus linear-model AIC. Negative ΔAIC values indicate better relative fit for the RCS specification. The general self-efficacy equation was adjusted for age, income, incidental physical activity duration, structured exercise frequency, structured exercise duration, wearable-device use, and ZTEx familiarity. The trigger equation additionally included general self-efficacy. The intention-proxy equation additionally included general self-efficacy and perceived physical activity triggers.

**Figure 1.** Restricted cubic spline associations between exercise motivation and variables in the adjusted ordered indirect-association model. Panel A presents the association between exercise motivation and general self-efficacy. Panel B presents the association between exercise motivation and perceived physical activity triggers after adjustment for general self-efficacy. Panel C presents the association between exercise motivation and the ZTEx-related intention proxy (ZTEx-IP) after adjustment for general self-efficacy and perceived physical activity triggers. Solid lines represent adjusted predicted values, and shaded areas represent 95% CIs. All models were adjusted for age, income, incidental physical activity duration, structured exercise frequency, structured exercise duration, wearable-device use, and ZTEx familiarity. The spline knots were located at exercise motivation scores of 33, 48, 54, and 72. ZTEx=zero-time exercise.

**Table 12.** Interval-specific direct and indirect associations in the nonlinear ordered-model robustness analysis

| **Exercise motivation contrast** | **Association** | **B** | **BootSE** | **95% bootstrap CI** |
| --- | --- | --- | --- | --- |
| P25 to P50 (44 to 51) | Direct association | 0.082 | 0.487 | -0.894 to 1.050 |
|  | Indirect association via general self-efficacy | 0.308 | 0.118 | 0.105 to 0.563 |
|  | Indirect association via perceived physical activity triggers | 1.039 | 0.196 | 0.675 to 1.449 |
|  | Ordered indirect association via self-efficacy and triggers | 0.099 | 0.038 | 0.037 to 0.186 |
|  | Total indirect association | 1.447 | 0.250 | 0.989 to 1.961 |
|  | Model-implied total association | 1.528 | 0.479 | 0.566 to 2.470 |
| P50 to P75 (51 to 56) | Direct association | -0.976 | 0.446 | -1.841 to -0.094 |
|  | Indirect association via general self-efficacy | 0.068 | 0.094 | -0.107 to 0.268 |
|  | Indirect association via perceived physical activity triggers | 0.898 | 0.175 | 0.577 to 1.261 |
|  | Ordered indirect association via self-efficacy and triggers | 0.022 | 0.031 | -0.034 to 0.089 |
|  | Total indirect association | 0.987 | 0.220 | 0.582 to 1.457 |
|  | Model-implied total association | 0.011 | 0.453 | -0.864 to 0.902 |
| P25 to P75 (44 to 56) | Direct association | -0.894 | 0.905 | -2.697 to 0.885 |
|  | Indirect association via general self-efficacy | 0.376 | 0.196 | 0.048 to 0.804 |
|  | Indirect association via perceived physical activity triggers | 1.937 | 0.366 | 1.259 to 2.697 |
|  | Ordered indirect association via self-efficacy and triggers | 0.121 | 0.063 | 0.017 to 0.265 |
|  | Total indirect association | 2.434 | 0.456 | 1.597 to 3.387 |
|  | Model-implied total association | 1.540 | 0.896 | -0.260 to 3.291 |

**Note.** N=790. B values are unstandardized interval-specific associations. BootSE=bootstrap standard error; CI=confidence interval. Percentile-based 95% CIs were estimated using 5000 nonparametric bootstrap resamples, all of which completed successfully. The model used 4-knot restricted cubic spline functions for exercise motivation and retained the same covariates as the primary PROCESS Model 6 analysis. Confidence intervals excluding 0 indicate statistically significant model-based associations. These estimates should not be interpreted as causal effects.

**Table 13.** Bootstrap comparisons of indirect associations between the lower and higher exercise motivation intervals

| Indirect association | Contrast | Difference | BootSE | 95% bootstrap CI |
| --- | --- | --- | --- | --- |
| Via general self-efficacy | P50-P75 minus P25-P50 | -0.240 | 0.087 | -0.423 to -0.084 |
| Via perceived physical activity triggers | P50-P75 minus P25-P50 | -0.142 | 0.066 | -0.273 to -0.015 |
| Ordered indirect association involving self-efficacy and triggers | P50-P75 minus P25-P50 | -0.077 | 0.028 | -0.138 to -0.028 |
| Total indirect association | P50-P75 minus P25-P50 | -0.459 | 0.120 | -0.701 to -0.228 |

**Note.** Negative values indicate that the indirect association was weaker for the increase from the 50th to the 75th percentile of exercise motivation than for the increase from the 25th to the 50th percentile. Confidence intervals were calculated from the within-resample differences between the 2 interval-specific associations using 5000 nonparametric percentile bootstrap resamples. All confidence intervals excluded 0.

6. **Secondary Exploratory Analysis: Occupational Subgroups**

To explore whether the association pattern differed by occupational category, the prespecified ordered indirect-association model was estimated separately among drivers and transport workers (n=112) and participants in other occupations (n=678). The same variables, covariates, and bootstrap procedure were used in both groups.

Among drivers and transport workers, the 95% bootstrap CIs for the total indirect association and the trigger-specific indirect association excluded 0, whereas those for the self-efficacy-specific and ordered indirect associations included 0. Among participants in other occupations, all specific and total indirect-association CIs excluded 0.

To formally compare occupational groups, a custom stratified bootstrap procedure (5,000 resamples) was implemented. Within each bootstrap sample, the PROCESS Model 6 regression equations were re-estimated separately for drivers/transport workers and participants in other occupations, and between-group differences were calculated for the direct association, each specific indirect association, the total indirect association, and the total association. The subgroup procedure reproduced the original PROCESS point estimates because the same regression equations were re-estimated; this was treated as a computational check rather than independent validation.

**Table 14.** Bootstrap comparison of direct and indirect associations between drivers/transport workers and participants in other occupations

| Association | Difference (Driver - Other) | BootSE | 95% bootstrap CI |
| --- | --- | --- | --- |
| Direct association | 0.012 | 0.148 | -0.288 to 0.302 |
| ZX → General self-efficacy → ZTEx-related intention proxy (ZTEx-IP) | 0.004 | 0.078 | -0.127 to 0.184 |
| ZX → Perceived physical activity triggers → ZTEx-related intention proxy (ZTEx-IP) | 0.007 | 0.091 | -0.173 to 0.184 |
| ZX → General self-efficacy → Perceived physical activity triggers → ZTEx-related intention proxy (ZTEx-IP) | 0.010 | 0.023 | -0.031 to 0.060 |
| Total indirect association | 0.021 | 0.109 | -0.190 to 0.241 |
| Total association | 0.033 | 0.116 | -0.204 to 0.256 |

**Note.** Difference = drivers/transport workers minus participants in other occupations. Positive values indicate larger associations among drivers/transport workers. Bootstrap confidence intervals were obtained from 5,000 stratified bootstrap resamples, with resampling performed independently within each occupational subgroup. Because all confidence intervals included zero, there was no statistically detectable evidence that the direct association, any specific indirect association, the total indirect association, or the total association differed between occupational groups.
