# Supplementary material for: Psychosocial Correlates of Sedentary Behavior Reduction Readiness Relevant to Zero-Time Exercise Among Chinese Adults in Sedentary Occupations: Cross-Sectional Study
Source: JMIR Public Health Surveill. 2026 Sep 4;12:e95707. doi: 10.2196/95707 (PMC13589206; doi:10.2196/95707)
Supplement: Supplementary file 6 [file publichealth_v12i1e95707_app6.docx]

**Multimedia Appendix 6. Multivariable-Adjusted Associations Between Exercise Motivation Tertiles and the ZTEx-IP Among Chinese Adults in Sedentary Occupations**

The lowest exercise motivation tertile was used as the reference group and is therefore not displayed. Points represent the adjusted B coefficients, and error bars represent 95% CIs. Compared with the lowest tertile, the adjusted B coefficients were 4.64 (95% CI 2.69-6.60) for the middle tertile and 8.49 (95% CI 6.43-10.60) for the highest tertile. A significant positive trend was observed across increasing exercise motivation tertiles (P for trend<.001). The model was adjusted for age, income, incidental physical activity duration, structured exercise frequency, structured exercise duration, wearable-device use, and ZTEx familiarity. B denotes the unstandardized regression coefficient.
