# Supplementary material for: Psychosocial Correlates of Sedentary Behavior Reduction Readiness Relevant to Zero-Time Exercise Among Chinese Adults in Sedentary Occupations: Cross-Sectional Study
Source: JMIR Public Health Surveill. 2026 Sep 4;12:e95707. doi: 10.2196/95707 (PMC13589206; doi:10.2196/95707)
Supplement: Supplementary file 7 [file publichealth_v12i1e95707_app7.docx]

**Multimedia Appendix 7. Factor Loadings From Harman’s Single-Factor Test Using All Core Questionnaire Items**

| Item | Factor loading | Communality, h² | Uniqueness, u² | Complexity |
| --- | --- | --- | --- | --- |
| BR1 | 0.410 | 0.168 | 0.832 | 1 |
| BR2 | 0.549 | 0.302 | 0.698 | 1 |
| BR3 | 0.413 | 0.171 | 0.829 | 1 |
| BR4 | 0.564 | 0.319 | 0.681 | 1 |
| BR5 | 0.171 | 0.029 | 0.971 | 1 |
| BR6 | 0.344 | 0.118 | 0.882 | 1 |
| BR7 | 0.533 | 0.284 | 0.716 | 1 |
| BR8 | 0.560 | 0.314 | 0.686 | 1 |
| BR9 | 0.180 | 0.033 | 0.967 | 1 |
| BR10 | 0.590 | 0.349 | 0.651 | 1 |
| BR11 | 0.368 | 0.136 | 0.864 | 1 |
| BR12 | 0.238 | 0.057 | 0.943 | 1 |
| BR13 | 0.527 | 0.278 | 0.722 | 1 |
| BR14 | 0.523 | 0.274 | 0.726 | 1 |
| BR15 | 0.593 | 0.352 | 0.648 | 1 |
| BR16 | 0.437 | 0.191 | 0.809 | 1 |
| BR17 | 0.608 | 0.369 | 0.631 | 1 |
| BR18 | 0.266 | 0.071 | 0.929 | 1 |
| SE1 | 0.533 | 0.285 | 0.715 | 1 |
| SE2 | 0.500 | 0.250 | 0.750 | 1 |
| SE3 | 0.566 | 0.320 | 0.680 | 1 |
| SE4 | 0.567 | 0.322 | 0.678 | 1 |
| SE5 | 0.574 | 0.330 | 0.670 | 1 |
| SE6 | 0.586 | 0.343 | 0.657 | 1 |
| SE7 | 0.604 | 0.365 | 0.635 | 1 |
| SE8 | 0.573 | 0.328 | 0.672 | 1 |
| SE9 | 0.560 | 0.313 | 0.687 | 1 |
| SE10 | 0.579 | 0.335 | 0.665 | 1 |
| PA1 | 0.604 | 0.365 | 0.635 | 1 |
| PA2 | 0.611 | 0.374 | 0.626 | 1 |
| PA3 | 0.608 | 0.370 | 0.630 | 1 |
| PA4 | 0.581 | 0.338 | 0.662 | 1 |
| PA5 | 0.550 | 0.302 | 0.698 | 1 |
| PA6 | 0.486 | 0.236 | 0.764 | 1 |
| PA7 | 0.570 | 0.325 | 0.675 | 1 |
| PA8 | 0.547 | 0.299 | 0.701 | 1 |
| PA9 | 0.573 | 0.328 | 0.672 | 1 |
| PA10 | 0.598 | 0.358 | 0.642 | 1 |
| PA11 | 0.515 | 0.266 | 0.734 | 1 |
| PA12 | 0.608 | 0.370 | 0.630 | 1 |
| PA13 | 0.600 | 0.360 | 0.640 | 1 |
| PA14 | 0.631 | 0.398 | 0.602 | 1 |
| AS1 | 0.711 | 0.506 | 0.494 | 1 |
| AS2 | 0.668 | 0.447 | 0.553 | 1 |
| AS3 | 0.680 | 0.463 | 0.537 | 1 |
| AS4 | 0.752 | 0.566 | 0.434 | 1 |
| AS5 | 0.747 | 0.558 | 0.442 | 1 |
| AS6 | 0.708 | 0.502 | 0.498 | 1 |
| AS7 | 0.724 | 0.525 | 0.475 | 1 |
| AS8 | 0.727 | 0.529 | 0.471 | 1 |
| AS9 | 0.686 | 0.471 | 0.529 | 1 |
| AS10 | 0.706 | 0.498 | 0.502 | 1 |
| AS11 | 0.737 | 0.544 | 0.456 | 1 |
| AS12 | 0.510 | 0.260 | 0.740 | 1 |
| AS13 | 0.464 | 0.215 | 0.785 | 1 |
| AS14 | 0.481 | 0.231 | 0.769 | 1 |
| AS15 | 0.491 | 0.241 | 0.759 | 1 |
| AS16 | 0.521 | 0.271 | 0.729 | 1 |

**Note.** Harman’s single-factor test was conducted using 58 core questionnaire items. BR1-BR18 were items from the Behavioral Regulation in Exercise Questionnaire-2 assessing exercise motivation; SE1-SE10 were items from the General Self-Efficacy Scale assessing general self-efficacy; PA1-PA14 were items from the Physical Activity Trigger Questionnaire assessing perceived physical activity triggers; and AS1-AS16 were items from the Adult Sedentary Behavior Reduction Intention Questionnaire assessing the ZTEx-related intention proxy (ZTEx-IP). One unrotated factor was extracted using principal axis factoring. Factor loading represents the standardized loading on the first unrotated factor. h² indicates communality, u² indicates uniqueness, and complexity reflects item complexity. The first factor explained 31.93% of the total variance.
