# Supplementary material for: Psychosocial Correlates of Sedentary Behavior Reduction Readiness Relevant to Zero-Time Exercise Among Chinese Adults in Sedentary Occupations: Cross-Sectional Study
Source: JMIR Public Health Surveill. 2026 Sep 4;12:e95707. doi: 10.2196/95707 (PMC13589206; doi:10.2196/95707)
Supplement: Supplementary file 8 [file publichealth_v12i1e95707_app8.docx]

**Multimedia Appendix 8. Adjusted Parallel Indirect-Association Model (PROCESS Model 4) Results**

This appendix presents detailed results from the adjusted parallel indirect-association model examining the association between exercise motivation and the ZTEx-related intention proxy involving general self-efficacy and perceived physical activity triggers.

**Table 1.** Adjusted regression coefficients for the parallel indirect-association model examining the association between exercise motivation and the ZTEx-related intention proxy (PROCESS Model 4).

| Outcome | Predictor | B | SE | t | P | 95% CI |
| --- | --- | --- | --- | --- | --- | --- |
| General self-efficacy | Exercise motivation | 0.214 | 0.015 | 13.876 | <.001 | 0.184 to 0.245 |
| General self-efficacy | Age | 0.067 | 0.173 | 0.387 | .699 | -0.273 to 0.407 |
| General self-efficacy | Income | 0.537 | 0.219 | 2.451 | .015 | 0.107 to 0.967 |
| General self-efficacy | Wearable device use | 0.160 | 0.368 | 0.435 | .664 | -0.562 to 0.881 |
| General self-efficacy | Incidental physical activity duration | 0.236 | 0.172 | 1.375 | .170 | -0.101 to 0.573 |
| General self-efficacy | Structured exercise frequency | 0.773 | 0.269 | 2.868 | .004 | 0.244 to 1.302 |
| General self-efficacy | Structured exercise duration | -0.036 | 0.237 | -0.150 | .881 | -0.500 to 0.429 |
| General self-efficacy | ZTEx familiarity | -0.218 | 0.330 | -0.662 | .508 | -0.865 to 0.429 |
| Perceived physical activity triggers | Exercise motivation | 0.585 | 0.031 | 19.045 | <.001 | 0.524 to 0.645 |
| Perceived physical activity triggers | Age | -0.682 | 0.344 | -1.982 | .048 | -1.358 to -0.007 |
| Perceived physical activity triggers | Income | -0.157 | 0.435 | -0.359 | .719 | -1.011 to 0.698 |
| Perceived physical activity triggers | Wearable device use | -2.447 | 0.731 | -3.349 | .001 | -3.881 to -1.013 |
| Perceived physical activity triggers | Incidental physical activity duration | 0.187 | 0.341 | 0.549 | .584 | -0.483 to 0.857 |
| Perceived physical activity triggers | Structured exercise frequency | 0.784 | 0.536 | 1.463 | .144 | -0.268 to 1.835 |
| Perceived physical activity triggers | Structured exercise duration | -0.189 | 0.470 | -0.402 | .688 | -1.112 to 0.734 |
| Perceived physical activity triggers | ZTEx familiarity | 2.749 | 0.655 | 4.195 | <.001 | 1.462 to 4.035 |
| ZTEx-related intention proxy | Exercise motivation | 0.167 | 0.040 | 4.150 | <.001 | 0.088 to 0.246 |
| ZTEx-related intention proxy | General self-efficacy | 0.483 | 0.075 | 6.428 | <.001 | 0.336 to 0.631 |
| ZTEx-related intention proxy | Perceived physical activity triggers | 0.311 | 0.038 | 8.239 | <.001 | 0.237 to 0.386 |
| ZTEx-related intention proxy | Age | 0.623 | 0.358 | 1.741 | .082 | -0.080 to 1.326 |
| ZTEx-related intention proxy | Income | 0.493 | 0.454 | 1.086 | .278 | -0.398 to 1.383 |
| ZTEx-related intention proxy | Wearable device use | -0.591 | 0.764 | -0.773 | .440 | -2.090 to 0.909 |
| ZTEx-related intention proxy | Incidental physical activity duration | 0.344 | 0.355 | 0.971 | .332 | -0.352 to 1.040 |
| ZTEx-related intention proxy | Structured exercise frequency | 0.799 | 0.559 | 1.431 | .153 | -0.298 to 1.896 |
| ZTEx-related intention proxy | Structured exercise duration | 0.733 | 0.488 | 1.503 | .133 | -0.224 to 1.690 |
| ZTEx-related intention proxy | ZTEx familiarity | -1.211 | 0.688 | -1.759 | .079 | -2.562 to 0.140 |

**Note.** PROCESS Model 4 was used to estimate the adjusted parallel indirect-association model. B values are unstandardized coefficients. The model was adjusted for age, income, wearable-device use, incidental physical activity duration, structured exercise frequency, structured exercise duration, and ZTEx familiarity. ZTEx = zero-time exercise; CI = confidence interval.

**Table 2.** Total, direct, and model-implied indirect associations between exercise motivation and the ZTEx-related intention proxy involving general self-efficacy and perceived physical activity triggers (adjusted PROCESS Model 4).

| Association component | Path | B | SE / BootSE | 95% CI |
| --- | --- | --- | --- | --- |
| Total association | Exercise motivation → ZTEx-related intention proxy | 0.453 | 0.034 | 0.385 to 0.520 |
| Direct association | Exercise motivation → ZTEx-related intention proxy | 0.167 | 0.040 | 0.088 to 0.246 |
| Total indirect association | — | 0.286 | 0.034 | 0.220 to 0.353 |
| Indirect association involving general self-efficacy | Exercise motivation → General self-efficacy → ZTEx-related intention proxy | 0.104 | 0.026 | 0.056 to 0.158 |
| Indirect association involving perceived physical activity triggers | Exercise motivation → Perceived physical activity triggers → ZTEx-related intention proxy | 0.182 | 0.026 | 0.129 to 0.233 |

**Note.** Indirect associations were estimated using 5000 percentile bootstrap samples. A 95% bootstrap CI excluding 0 was treated as statistical evidence of a nonzero indirect association. B values are unstandardized coefficients. The model was adjusted for age, income, wearable-device use, incidental physical activity duration, structured exercise frequency, structured exercise duration, and ZTEx familiarity. ZTEx = zero-time exercise; CI = confidence interval.
